# Galactoside-Based Molecule Enhanced Antimicrobial Activity through Acyl Moiety Incorporation: Synthesis and In Silico Exploration for Therapeutic Target

**DOI:** 10.3390/ph16070998

**Published:** 2023-07-13

**Authors:** Faez Ahmmed, Samiah Hamad Al-Mijalli, Emad M. Abdallah, Ibrahim H. Eissa, Ferdausi Ali, Ajmal R. Bhat, Joazaizulfazli Jamalis, Taibi Ben Hadda, Sarkar M. A. Kawsar

**Affiliations:** 1 Laboratory of Carbohydrate and Nucleoside Chemistry, Department of Chemistry, Faculty of Science, University of Chittagong, Chittagong 4331, Bangladesh; faezahmed8@gmail.com; 2 Department of Biology, College of Sciences, Princess Nourah Bint Abdulrahman University, Riyadh 11671, Saudi Arabia; shalmejale@pnu.edu.sa; 3 Department of Science Laboratories, College of Science and Arts, Qassim University, Ar Rass 51921, Saudi Arabia; 140208@qu.edu.sa; 4 Pharmaceutical Medicinal Chemistry & Drug Design Department, Faculty of Pharmacy (Boys), Al-Azhar University, Cairo 116884, Egypt; ibrahimeissa@azhar.edu.eg; 5 Department of Microbiology, Faculty of Biological Science, University of Chittagong, Chittagong 4331, Bangladesh; seema@cu.ac.bd; 6 Department of Chemistry, RTM Nagpur University, Nagpur 440033, India; bhatajmal@gmail.com; 7 Department of Chemistry, Universiti Teknologi Malaysia, Johor Bahru 81100, Malaysia; joazaizulfazli@utm.my; 8 Laboratory of Applied Chemistry & Environment, Faculty of Sciences, Mohammed Premier University, Oujda 60000, Morocco; taibi.ben.hadda@gmail.com

**Keywords:** galactosides, dengue virus, antimicrobial, molecular docking, dynamics, ADMET

## Abstract

In this study, a series of galactoside-based molecules, compounds of methyl *β*-d-galactopyranoside (MDGP, **1**), were selectively acylated using 2-bromobenzoyl chloride to obtain 6-*O*-(2-bromobenzoyl) substitution products, which were then transformed into 2,3,4-tri-*O*-6-(2-bromobenzoyl) compounds (**2**–**7**) with various nontraditional acyl substituents. The chemical structures of the synthesized analogs were characterized by spectroscopic methods and physicochemical and elemental data analyses. The antimicrobial activities of the compounds against five human pathogenic bacteria and two phyto-fungi were evaluated in vitro and it was found that the acyl moiety-induced synthesized analogs exhibited varying levels of antibacterial activity against different bacteria, with compounds **3** and **6** exhibiting broad-spectrum activity and compounds **2** and **5** exhibiting activity against specific bacteria. Compounds **3** and **6** were tested for MIC (minimum inhibitory concentration) and MBC (minimum bactericidal concentration) based on their activity. The synthesized analogs were also found to have potential as a source of new antibacterial agents, particularly against gram-positive bacteria. The antifungal results suggested that the synthesized analogs could be a potential source of novel antifungal agents. Moreover, cytotoxicity testing revealed that the compounds are less toxic. A structure-activity relationship (SAR) investigation revealed that the lauroyl chain [CH_3_(CH_2_)_10_CO-] and the halo-aromatic chain [3(/4)-Cl.C_6_H_4_CO-] in combination with sugar, had the most potent activity against bacterial and fungal pathogens. Density functional theory (DFT)-calculated thermodynamic and physicochemical parameters, and molecular docking, showed that the synthesized molecule may block dengue virus 1 NS2B/NS3 protease (3L6P). A 150 ns molecular dynamic simulation indicated stable conformation and binding patterns in a stimulating environment. In silico ADMET calculations suggested that the designed (MDGP, **1**) had good drug-likeness values. In summary, the newly synthesized MDGP analogs exhibit potential antiviral activity and could serve as a therapeutic target for dengue virus 1 NS2B/NS3 protease.

## 1. Introduction

There is a pressing need for the development of new antimicrobial drugs to combat the growing threat of antimicrobial resistance. New drugs are needed to target resistant bacteria, as well as to provide alternative treatments for common infections. However, the development of new antimicrobial drugs is challenging and the pipeline for new drugs is limited [1,2]. Organic chemistry plays a critical role in the innovation of new antimicrobial drugs. Most antimicrobial drugs are organic molecules that are intended to target specific parts of microbial cells, such as cell walls or enzymes [3]. Chemical synthesis is used to make and change these molecules, which improves their pharmacokinetic qualities, makes them more effective, and makes them less harmful. Additionally, chemical biology is a key part of finding new drug targets and making lead compounds work better. Using methods from chemical science to design and make new scaffolds can lead to the production of entirely new classes of antimicrobial drugs. In general, organic synthesis is a very important part of finding and making new antibacterial drugs [4].

Carbohydrates, the most available organic biomolecules, remain an attractive research subject for scientists due to their vital role in biological systems. The roles of carbohydrates comprise cell proliferation, communication between cells, and the immune response [5]. It is also the main source of metabolic energy and fine-tunes cell–cell connections and other key biological processes [6,7]. Carbohydrates also suppress harmful bacteria, viruses, protozoa, and fungi [8]. Due to their antimicrobial properties, carbohydrates have also been used to treat infectious disorders [9]. To synthesize novel antimicrobial agents with enhanced activity, a deep understanding of the antimicrobial action mechanism is best. The dengue virus (DENV), a member of the *Flaviviridae* family, is the most common arthropod-transmitted virus in humans. It causes self-limiting dengue fever, potentially fatal dengue hemorrhagic fever (DHF), and dengue shock syndrome (DSS) [10,11]. Within the genus Flavivirus, four closely related viral serotypes are associated with other human disease-causing viruses such as West Nile virus (WNV), yellow fever virus (YFV), and Japanese encephalitis virus (JEV).

MDGP derivatives can form a large group of natural proteins and synthetic agents that can strongly interact with glycosylated proteins. MDGP derivatives can be synthesized and isolated from different organisms. Each carbohydrate binding agent interacts in a specific way with monosaccharides, such as mannose, glucose, and galactose, residues present in the backbone of *N*-glycan structures. Since many enveloped viruses are glycosylated at the viral surface, such as HIV, HCV, and DENV, MDGP derivatives can interact with the glycosylated envelope of the virus and subsequently prevent viral entry into the host cell. Previously, antiviral activity against HIV and HCV was demonstrated for several carbohydrate binding agents isolated from plants and algae specifically binding mannose and *N*-acetylglucoosamine.

A literature survey suggested that biologically active molecules consist of aromatic ring substituents [12,13]. Benzene and its homologs, as well as nitrogen, sulfur, and halogen, act as a group and are known to enhance the biological activity of the parent molecule [14,15,16]. Moreover, it was reported that merging two active nuclei to form a molecule also increased the biological efficacy of the molecule [17,18]. The combination of two or more heteroaromatic rings and acyl groups can also increase the biological activity of the parent nucleus [19,20]. Monosaccharide derivatives have broad-spectrum antibacterial action against gram-negative and gram-positive pathogens, such as *Escherichia coli*, *Bacillus subtilis*, *Salmonella typhi*, and *Staphylococcus aureus* [21]. Monosaccharide analogs inhibited cancer cells in a recent study [22]. Isosteric changes at the hydroxyl group of nucleoside and monosaccharide structures were used to produce powerful antiviral [23,24,25] and antibacterial drugs [26]. Based on the above findings, we sought novel antimicrobial agents [27,28]. The current study aimed to synthesize a panel of (MDGP, **1**) analogs (**2**–**7**) and evaluate their in vitro antimicrobial activity against eight pathogens. Additionally, the study aimed to perform molecular docking studies against dengue virus 1 NS2B/NS3 protease (3L6P) and report the prediction of activity spectra for substances (PASS). To confirm the stability of the docked complexes, molecular dynamic simulations were performed for 150 ns. Furthermore, the study focused on optimizing the synthesized (MDGP, **1**) compounds and investigating their physicochemical behavior through density functional theory (DFT) studies. The overall objective of the study was to evaluate the potential of the synthesized analogs as antimicrobial and antiviral agents.

## 2. Results and Discussion

### 2.1. Chemistry

**Scheme 1:**
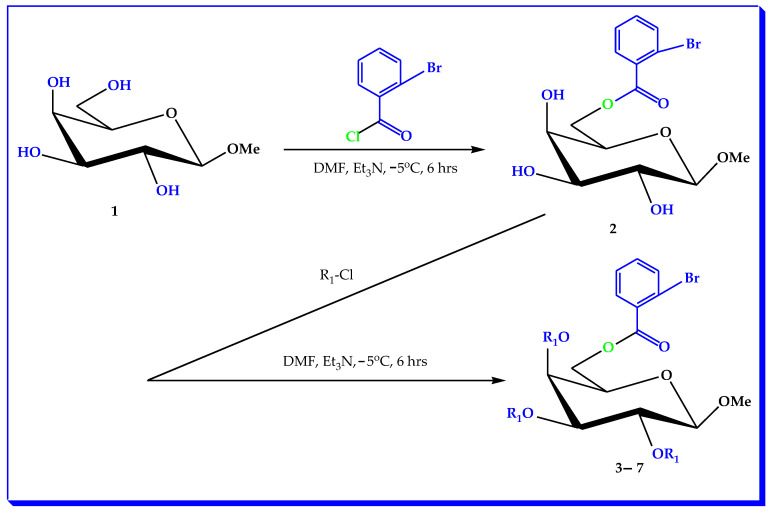
General procedure for synthesizing compounds **2** to **7** of (MDGP, **1**).

**Figure 1:**
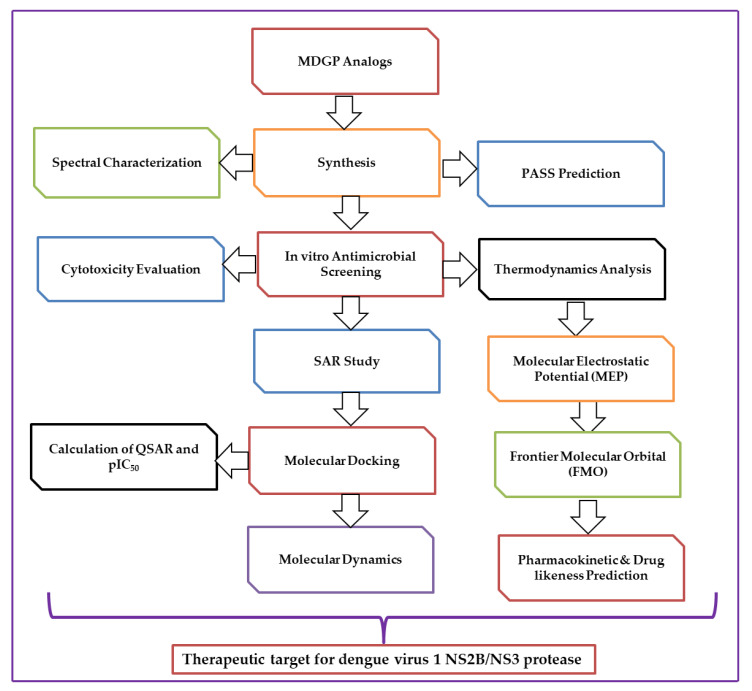
Illustrates the workflow of the present study.

The main objective of this research work is to achieve the selective bromobenzoylation (Scheme 1) of MDGP (**1**) with 2-bromobenzoyl chloride using a direct acylation method. The resulting 2-bromobenzoylation product was transformed into a number of compounds employing various aliphatic and aromatic agents (Table 1). Figure 1 shows a schematic flow of the work plan.

### 2.2. Characterization

Regioselective 2-bromobenzoylation of methyl *β*-d-galactopyranoside (**1**) with 2-bromobenzoyl chloride utilizing the direct method was the initial goal of this work. A series of derivatives of the 2-bromobenzoylation product were acylated with six different acylating agents. Compound **2** was obtained as needles at 101–102 °C in 92% yield after conventional work-up and purification. The 2-bromobenzoyl derivative (**2**)’s structure was determined by ^1^H NMR and ^13^C NMR and mass spectra; (C=O) and (br, –OH) stretching absorption peaks were observed in this compound’s FTIR (Figure S1). In its ^1^H NMR spectrum (Figure S2), the presence of one 2-bromobenzoyl group in the molecule was confirmed by observing the following peaks: two one-proton doublets at δ 7.81 (as d, Ar-H) and δ 7.63 (as d, Ar-H) and a two-proton multiplet at δ 7.31 (1H, Ar-H) corresponding to the aromatic ring protons of the molecule. The 2-bromobenzoyl group was introduced at position 6 when C-6 deshielded from its usual value (~4.00 ppm) to 4.52 (as dd, J = 11.0, 6.4 Hz, 6a) and 4.50 (as dd, J = 11.0, 6.6 Hz, 6b) [29]. Derivative (**2**) may arise because of the increased reactivity of the sterically less hindered primary -OH group, 1-OH > 2-OH > 3-OH. The ^13^C NMR spectra showed all the telltale peaks of a palmitoyl group. The chemical formula of compound (**3**) was C_14_H_17_O_7_Br and its mass spectrum featured a molecular ion peak at m/z [M + 1]^+^ 378.14.

### 2.3. Two-Dimensional NMR

**Figure 2:**
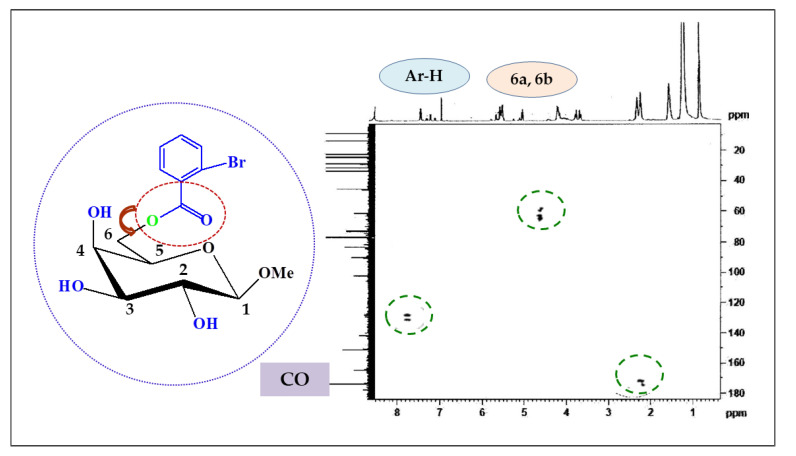
The HMBC correlations of analog **2**; -CO with Ar-H, H-6b, and H-6b protons.

Attribution of the signals by analyzing its COSY, HSQC, and HMBC spectral experiments (Table 2 and Figure 2), along with the ^13^C NMR spectrum, ascertained the structure as methyl 6-*O*-(2-bromobenzoyl)-*β*-d-galactopyranoside (**2**).

The 2,3,4-tri-*O*-lauroyl compound (**3**) was prepared to support the structure of the 6-*O*-(2-bromobenzoyl) compound (**2**). Thus, treatment of the 2-bromobenzoate (**2**) with lauroyl chloride in pyridine yielded the laurate (**3**) in 90% yield. Two six-proton multiplets at δ 2.36 and δ 1.65 {3 × CH_3_(CH_2_)_9_CH_2_CO-}, a forty-eight-proton multiplet at δ 1.27, and a nine-proton multiplet at δ 0.89 were due to the three lauroyl groups. The three lauroyl groups were introduced at positions 2, 3, and 4 because the C-2, C-3, and C-4 protons were deshielded to δ 4.96 (as m), δ 4.91, and δ 4.51 from their predecessor (compound **2**) values of δ 3.87, δ 4.00, and δ 4.19. The structure of this molecule was determined by analyzing the FTIR, ^1^H NMR, ^13^C NMR, and mass spectra (Table 3) [30].

Again, the reaction of compound **2** with an excess of myristoyl chloride in pyridine, followed by aqueous work-up and chromatographic purification, yielded the myristoyl derivative (**4**) as needles, mp. 154–155 °C. After spectroscopic examination, we determined this compound’s structure as methyl 6-*O*-(2-bromobenzoyl)-2,3,4-tri-*O*-myristoyl-*β*-d-galactopyranoside (**4**). 3-Chlorobenzoyl chloride was utilized to directly acylate molecule **2**. We obtained the 3-chlorobenzoyl derivative (**5**) after the typical work-up and purification. The structures of these compounds were confidently assigned as methyl 6-*O*-(2-bromobenzoyl)-2,3,4-tri-*O*-(3-chlorobenzoyl)-*β*-d-galactopyranoside (**5**) based on their spectra, which showed characteristic peaks at δ 8.01 (3H, m, Ar-H), δ 7.82 (3H, m, Ar-H), δ 7.47 (3H, m, Ar-H), and δ 7.34 (3H, m, Ar-H). Similar techniques were used to isolate compound **6**. In its ^1^H NMR spectra, p-substituted benzoyl groups have two six-aromatic proton multiplets at δ 8.01 (as Ar-H) and δ 7.81 (as Ar-H). Conversion to 4-*t*-butylbenzoate (**7**) confirmed the structure of 6-*O*-2-bromobenzoate (2). The molecule’s ^1^H NMR spectra showed three 4-*t*-butylbenzoyl groups as two six-proton multiplets at δ 8.04 and δ 7.59 (3 × Ar-H) and three singlets at δ 1.25, δ 123, and δ 122 {27H, 3 × s, 3 × (CH_3_)_3_C-}. The rest of the spectra were consistent with the structure reported as methyl 6-*O*-(2-bromobenzoyl)-2,3,4-tri-*O*-(4-*t*-butylbenzoyl)-*β*-d-galactopyranoside (**7**).

### 2.4. Antibacterial Susceptibility

The results of the antibacterial screening of the tested synthesized compounds are shown in Table 4 and Figures S2 and S3. In general, the antibacterial activity of the compound is divided into three categories: weak activity at 10 mm or less, moderate activity at 10 to 15 mm, and high activity at 15 mm or more [31,32,33]. Accordingly, the results presented in this study demonstrate the antibacterial activity of seven synthesized analogs against a range of bacteria. Among the tested analogs, compounds **3** and **6** exhibited wide-spectrum antibacterial activity against all tested bacteria, with compound **3** showing particularly high activity against *B. cereus*. Compound **2** also showed antibacterial activity against most bacteria tested, except for *B. subtilis* but it demonstrated high activity against *P. aeruginosa*. Compound **5** showed no activity against any tested bacteria except for *P. aeruginosa*. On the other hand, compounds **1** and **4** did not exhibit any antibacterial activity and compound **7** showed weak or no activity against all tested bacteria. These results are consistent with previous studies that have reported the antibacterial activity of similar compounds against different bacterial strains. For example, compounds **3** and **6** have structural similarities to known antibacterial agents, such as macrolides and ketolides, which have been shown to have wide-spectrum activity against both gram-positive and gram-negative bacteria [34,35]. Compound **2**, which showed high activity against *P. aeruginosa*, has a structure similar to tetracyclines, which are known to be effective against this bacterial strain. Compound **5**, which showed no activity against most bacteria tested except for *P. aeruginosa*, may have a specific mechanism of action that targets this bacterial strain. Previous studies have shown that *P. aeruginosa* is resistant to many antibiotics due to its ability to form biofilms, which can protect it from antimicrobial agents [36]. Therefore, the high activity of compound **5** against *P. aeruginosa* suggests that it may have a unique mode of action that can overcome the resistance mechanisms of this bacterial strain. The observation that the synthesized compounds showed superior activity against gram-positive bacteria compared to gram-negative bacteria is consistent with previous studies that have reported similar findings [37]. This may be due to the differences in the cell-wall structure between gram-positive and gram-negative bacteria, which can affect the penetration and efficacy of antibacterial agents [38] (Figure S4). By inhibiting these fundamental pathways, glucopyranoside analogs are often efficient antibacterial agents [39].

### 2.5. MIC and MBC Measurement

**Figure 3:**
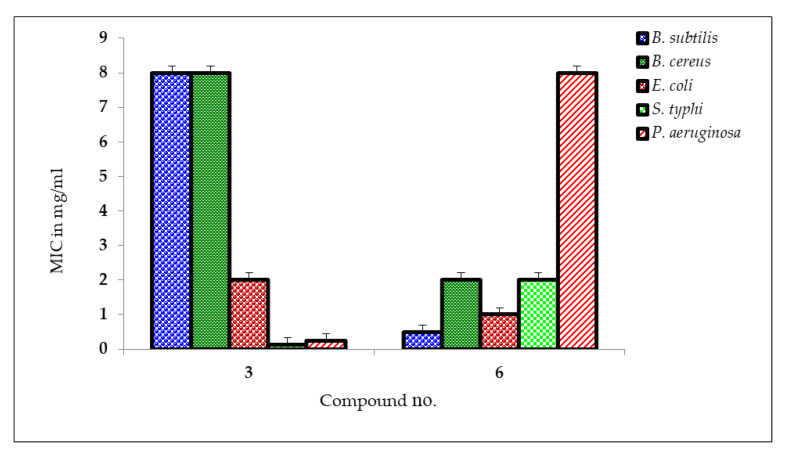
MIC values of compounds **3** and **6** against the tested organisms.

**Figure 4:**
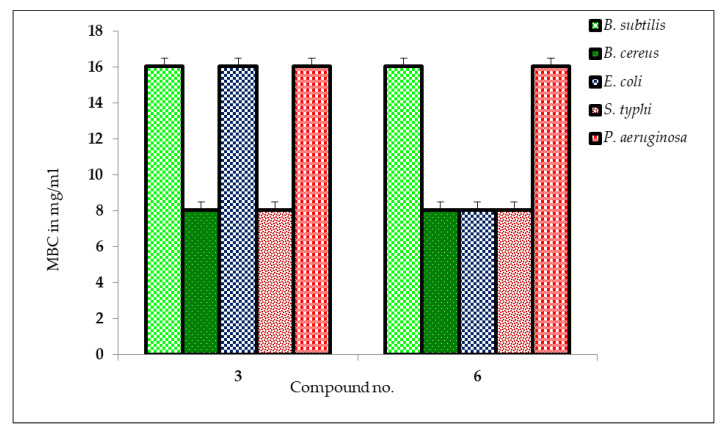
MBC values of analogs **3** and **6** against the tested organisms.

In this study, the antibacterial activity of two methyl *β*-D-galactopyranoside analogs (**3** and **6**) was tested using MIC and MBC assays (Figure 3 and Figure 4 and Table S1). The disc-diffusion test indicated that these analogs had strong antibacterial activity, which prompted further investigation. The MIC values (Figure 3) revealed that lower MIC values corresponded to greater antimicrobial activity, indicating that drugs with lower MIC scores are more effective against bacterial growth [40]. Compound **3** showed the lowest MIC value with *S. typhi* (0.125 mg/mL), followed by *P. aeruginosa* (0.25 mg/mL) and *E. coli* (2.0 mg/mL), and the highest MIC values (less susceptibility) with *B. subtilis* and *B. cereus* (8.0 mg/mL). Compound **6** showed the lowest MIC values (the most susceptible) with *B. subtilis* (0.5 mg/mL), followed by *E. coli* (1.0 mg/mL), *B. cereus*, and *S. typhi* (2.0 mg/mL), while the highest MIC value (low susceptibility) was recorded by *P. aeruginosa* (8.0 mg/mL). Previous studies have also investigated the antibacterial activity of *β*-galactoside analogs. For example, it was found that *ortho*-nitrophenyl-*β*-galactoside analogs had remarkable antibacterial activity [41].

Similarly, a published study [42,43] reported that a methyl-(3-(1-(3,4-dichlorophenyl)-1H-1,2,3-triazol-4-yl)methoxy)phenyl)-*β*-carboline-3-car-boxylate analog exhibited antibacterial activity against various pathogenic bacteria with low MIC values (ranging between 64 and >128 µg/mL). These findings suggest that *β*-galactoside analogs are promising antimicrobial agents with low MIC values and the results of the present study further support this notion.

On the other hand, the results of the MBC test, which indicated the minimal antibacterial chemical concentration required to eradicate 99.9% of the test organisms from the original inoculum, are shown in Figure 4. The results of the MBC test revealed that *B. cereus* and *S. typhi* were the most susceptible bacteria to both analogs (methyl *β*-d-galactopyranoside compounds **3** and **6**), requiring only 8.0 mg/mL to eradicate 99.9% of the test organisms from the original inoculum. On the other hand, *B. subtilis*, *E. coli*, and *P. aeruginosa* required 16.0 mg/mL of the analogs to achieve the same level of antibacterial activity, according to the MBC test. The findings of this study are consistent with previous studies that have demonstrated the antibacterial potential of methyl *β*-d-galactopyranoside analogs against various bacterial species. For example, previous studies have investigated the antibacterial activity of a series of methyl *β*-d-galactopyranoside analogs against methicillin-resistant *S. aureus* (MRSA) and found that some analogs showed potent activity against MRSA, with low MBC values [44,45]. Moreover, the antibacterial activity of a methyl *β*-D-galactopyranoside derivative against *E. coli* was studied, and the compound exhibited a dose-dependent bactericidal effect against bacteria with MBC values ranging from 0.704 to 1.408 mg/mL [27]. In conclusion, the results of this study indicate that methyl *β*-d-galactopyranoside compounds **3** and **6** possess antibacterial activity against a range of bacterial species, with *B. cereus* and *S. typhi* being the most susceptible strains. Further research is warranted to investigate the potential clinical applications of these analogs in treating bacterial infections in vivo using experimental animals.

### 2.6. Antifungal Potential

Antifungal activity is an essential feature of a compound, especially in the treatment of fungal infections in agricultural activities and disease after harvest [46]. In this study, the antifungal activity of methyl *β*-d-galactopyranoside compounds was investigated against two phyto-fungal strains and the results were compared with those of the standard antibiotic nystatin. Table 5 presents the percent inhibition of the growth of the test fungal organisms by the tested methyl *β*-d-galactopyranoside compounds and the standard antibiotic nystatin. The results indicate that all the test compounds were sensitive toward the mycelial growth of fungi at different levels. Notably, three test compounds (**2**, **3**, and **6**) exhibited very high effectiveness against all the fungal strains used and, in most cases, the inhibition was higher than that of the standard antibiotic nystatin (Figure S5). This finding suggests that the tested analogs could be potential antifungal agents. In addition, compound **7** showed high inhibition against *A. niger*, while no inhibition was observed against *A. flavus*. This result suggests that the antifungal activity of the analogs varies depending on the fungal strain. This observation is consistent with previous studies that have reported the differential susceptibility of fungal strains to antifungal agents [47]. Moreover, the acylation of methyl *β*-d-galactopyranoside improves antifungal activity. This finding is consistent with previous studies that have reported the modification of sugar moieties as a strategy to improve the antifungal activity of compounds [48]. Therefore, the results of this study suggest that methyl *β*-d-galactopyranoside analogs could be a potential source of novel antifungal agents. Comparing the results of this study with those of previous studies, it is worth noting that the tested analogs exhibited high effectiveness against all the fungal strains used, which is a promising finding. However, further studies are necessary to investigate the mechanism of action of these analogs and their potential toxicity in vivo. Additionally, future studies could explore the potential of combining these analogs with other antifungal agents to improve their efficacy.

### 2.7. Cytotoxic Activity of MDGP Compounds

**Figure 5:**
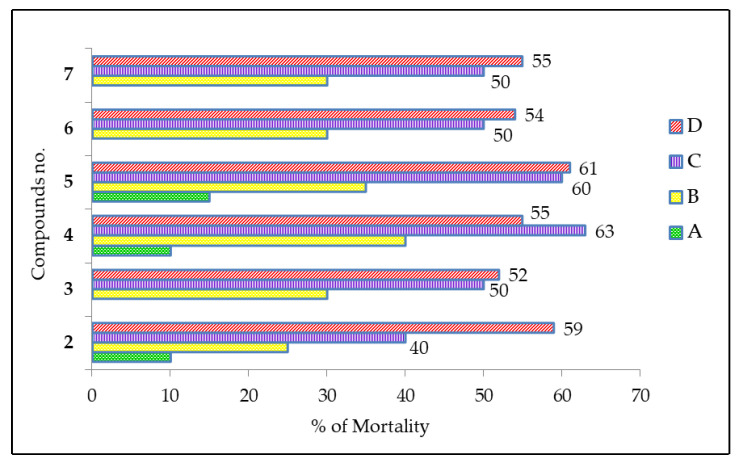
Cytotoxic activity of uridine derivatives (**2**–**7**).

Figure 5 displays the cytotoxicity of synthesized MDGP compounds (**2**–**7**) as assessed by the brine shrimp lethality bioassay method [49]. The figure depicts the mortality percentage of shrimp at 24 and 48 h. The hydrophobicity and cytotoxicity were enhanced by long alkyl chains and phenyl rings, as reported in [44]. MDGP compound **3** (methyl 6-*O*-(2-bromobenzoyl)-2,3,4-tri-*O*-lauroyl-*β*-d-galactopyranoside) exhibited the least toxicity, resulting in a mortality rate of 30.09%, as per the data analysis. Compounds **4** (methyl 6-*O*-(2-bromobenzoyl)-2,3,4-tri-*O*-myristoyl-*β*-d-galactopyranoside) and **5** (methyl 6-*O*-(2-bromobenzoyl)-2,3,4-tri-*O*-(3-chlorobenzoyl)-*β*-d-galactopyranoside) exhibited the highest toxicity levels, resulting in increased mortality of 37.11% to 38.17%. This observation indicates that benzoyl derivatives exhibit lower cytotoxicity than alkyl chain derivatives. Furthermore, the cytotoxicity of alkyl chain derivatives exhibits a positive correlation with concentration.

### 2.8. Structure–Activity Relationship

**Figure 6:**
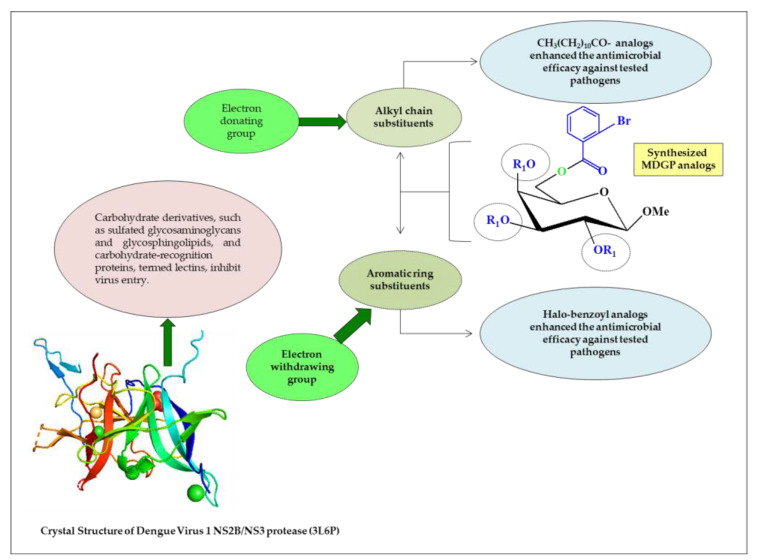
Structure–activity relationship of the synthesized (MDGP, **1**) compounds.

Antimicrobial agents are used to prevent infections and diseases caused by pathogens and are heterocyclic molecules that play a vital role in the metabolism of living cells [50,51]. Structure–activity relationship (SAR) scanning is important for understanding the mechanisms of antibacterial activity for MDGP analogs (Figure 6). The SAR of MDGP analogs can be seen from the results of the antimicrobial activities displayed in Table 4 and Table 5. Starting molecule **1** itself showed no activity against infective bacteria, so a swap in the **1** skeleton greatly affected the antibacterial activity. For most of the tested bacteria, fused lauroyl, 4-chlorobenzoyl moieties were more active than the 2-bromobenzoyl moiety. In contrast, the lauroyl containing compound (**3**) was stronger than the myristoyl compound (**4**). When aromatic-, alkyl- or electron-withdrawing groups are attached to the parent molecule, the antimicrobial activities are increased. In addition, hydrophobicity also contributes to increasing the antimicrobial activity. When hydrophobic interactions might occur between acyl chains of MDGP, **1** accumulates in the lipid-like nature of the bacterial membranes. Due to their hydrophobic interactions, bacteria lose their membrane permeability and, consequently, die [52].

**Figure 7:**
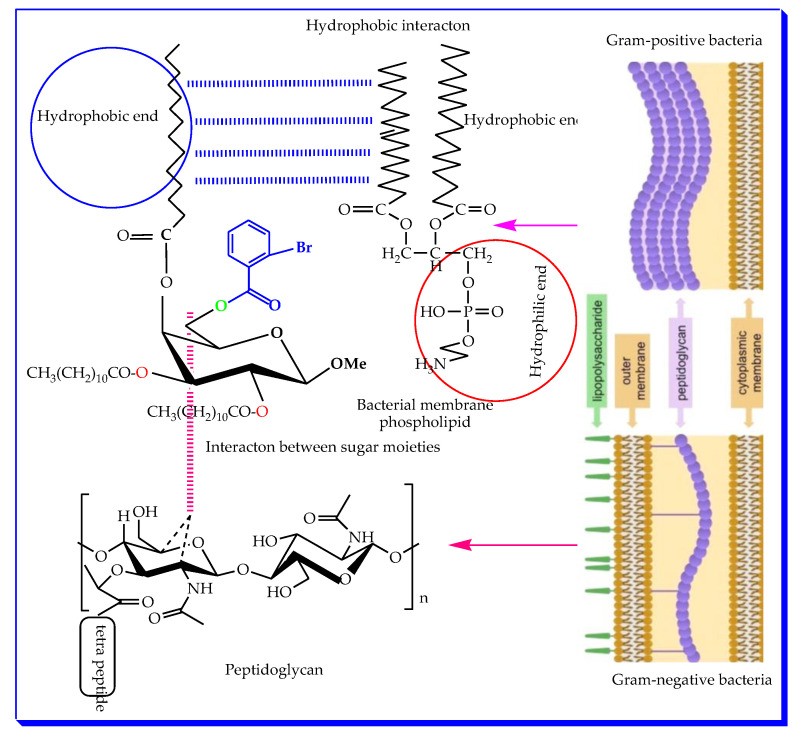
SAR study of the MDGP compounds with bacterial pathogens.

The acyl chains of MDGP accumulated in the lipid-like structure of bacterial membranes are hypothesized to engage in analogous hydrophobic interactions. Hydrophobic contact causes bacteria to lose membrane permeability, which leads to organism death. The structure–activity relationship (SAR) of the synthesized MDGP compounds was attempted based on the results. It was also shown that the antibacterial activity of MDGP (**1**) was enhanced by the addition of 2-Br.C_6_H_5_CO- and CH_3_(CH_2_)_10_CO-groups to either the C-6 position or the C-2, C-3, and C-4 positions of compounds **1** or **2** (Figure 7). In addition, a computational study of the synthesized MDGP analogs revealed promising binding affinity against the membrane and NS2B/NS3 protease. Carbohydrate derivatives resemble natural glucoside/galactoside and incorporate into DNA and RNA to facilitate cellular metabolism. Most antibiotic carbohydrate derivatives work by obstructing viral DNA or RNA polymerase during the chain extension step of replication.

### 2.9. In Silico Studies

In silico studies are very popular and informative and are frequently used to predict how a compound will react with targeted proteins with ligands. In silico studies were performed by the following parameters.

### 2.10. Assessment of Antimicrobial Activity by PASS and Bioactivity

PASS predicted the antibacterial spectrum of compounds **2**–**7** (MDGP, **1**). The PASS results are shown in Table 6 as Pa and Pi. The results showed that (MDGP, **1**) compounds **2**–**7** have 0.48 < Pa < 0.55 antibacterial, 0.44 < Pa < 0.56 antifungal, and 0.39 < Pa < 0.60 antiviral activity. These compounds were more effective against bacterial and viral infections than fungal species. The insertion of lauroyl and myristoyl substituted groups decreased the antibacterial activity of (MDGP, **1**) (Pa = 0.414), while the addition of the *t*-butylbenzoyl group boosted it (Pa = 0.551). The most antifungal was compound **6**, which had a halo-benzoyl aromatic group.

The bioactivity score of molecules is greater than 0.00 if they have promising biological activity, 0.50 to 0.00 if they are moderately active, and −0.50 if they are inactive. Table 7 shows the bioactivity scores of all designed (MDGP, **1**) compounds. The bioactivity score showed promising efficacy for compounds **2**, **3**, and **5–7**.

### 2.11. Thermodynamic Analysis

Compound **5** has the greatest free energy (−6025.426 Hartree). The maximum enthalpy (−6025.296 Hartree) and electronic energy (−6025.295 Hartree) were also found in it. A high dipole moment value indicates polarity [53,54]. As demonstrated in Table S2, three compounds (**3**, **4**, and **6**) have an elevated dipole moment, which makes them more polar and promotes nonbonding interactions with the receptor protein. Compound (**6**)’s bulky group gave it the largest dipole moment (8.200 Debye), suggesting greater binding affinity. As the number of carbon atoms grew and the substituents had aromatic rings (**2**–**7**), all criteria scored higher. Thus, the MDGP compounds’ thermodynamic characteristics are greatly enhanced by the acylation of their (-OH) groups.

### 2.12. Frontier Molecular Orbital (FMO)

Electronic absorption is the transition from the ground to the first excited state and is generally defined by one electron excitation from the HOMO to LUMO [55]. Table 8 shows the frontier molecular orbital (FMO) index of all tested compounds.

**Figure 8:**
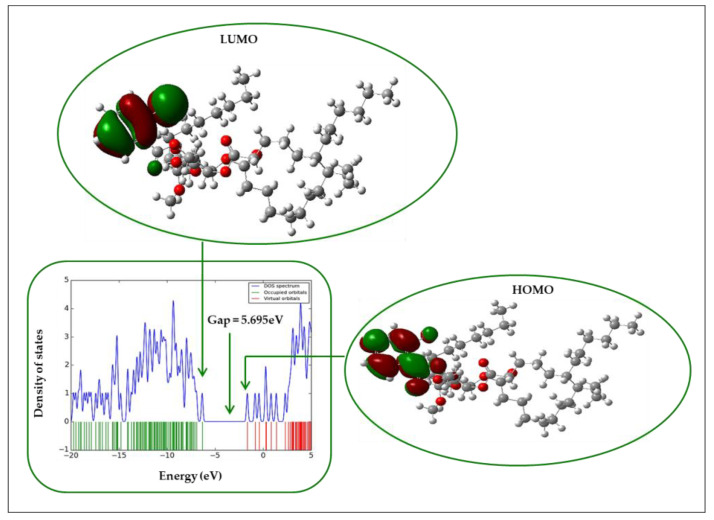
DOS plot and HOMO-LUMO energy gap of compound **4**.

As indicated in Table 8 and Figure 8, compound **4** had a higher energy-gap value (5.692 eV) than the other analogs, whereas compound **5** had a somewhat lower value (4.945 eV). Compound **5** has the lowest gap (4.945 eV) and maximum softness (0.404 eV) (Figure 8).

### 2.13. Molecular Electrostatic Potential (MEP)

**Figure 9:**
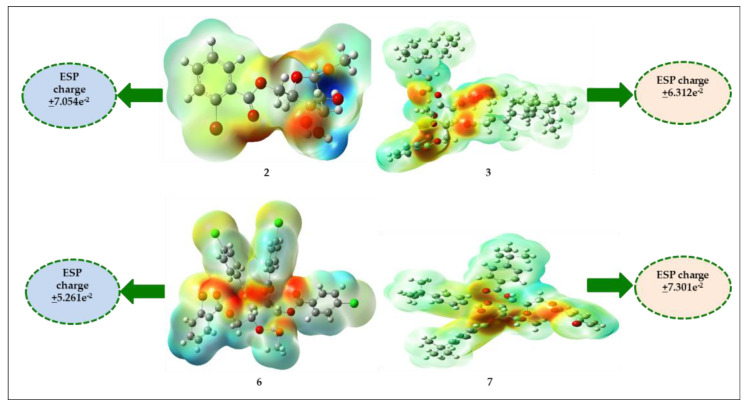
Molecular electrostatic potential (MESP) map of (MDGP, **1**) compounds (**2**–**7**).

The molecular electrostatic potential (MEP) predicted reactive sites for the electrophilic and nucleophilic assault of all organic compounds [56,57]. Color-grading MEPs show molecular size, shape, and positive, negative, and neutral electrostatic potential areas. The MEP predicted electrophilic and nucleophilic reactive sites of MDGP, **1**, and its derivatives (**2**–**7**). Red is the maximum negative area, which is good for electrophilic assault, blue is the maximum positive area, good for a nucleophilic attack, and green is the zero potential region (Figure 9).

### 2.14. Molecular Docking

**Figure 10:**
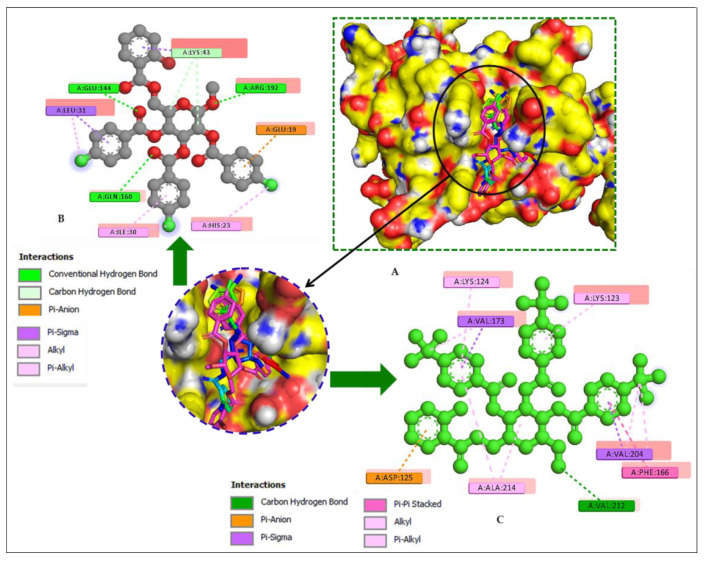
(**A**) Docking pose (space-filling model) and 2D interaction map of compounds **6** and **7** with dengue virus-1 NS2B/NS3 protease, (**B**) Nonbonding interactions of compound **6** with the active site of dengue virus-1 NS2B/NS3 protease, (**C**) Nonbonding interactions of compound **7** with the active site of dengue virus-1 NS2B/NS3 protease.

In this study, AutoDock Vina software was used to study the binding energy and interaction modes of a series of compounds (MDGP, **1**) with the dengue virus-1 NS2B/NS3 protease (3L6P) active site (Table 9 and Table 10). Docking screening revealed six compounds (**2**–**7**) with the highest binding energies to define the probable (MDGP, **1**) compound binding behavior. Aromatic compounds have superior binding scores than aliphatic analogs, as indicated in Table 9. Figure 8 depicts the docked conformation of the most active molecules (**6** and **7**) based on docking studies. The results (Figure 10) showed that (MDGP, **1**) compounds (**2**–**7**) are the most promising ligands with binding energies of −6.6, −6.1, −5.1, −8.1, −8.0, and −8.3 kcal/moL, respectively. These compounds are bound with both proteins via many hydrophobic bonding and hydrogen interactions. The binding sites were mainly located in a hydrophobic cleft bordered by the amino acid residues Ile215, Ile30, Val204, Val173, Ala141, Ala214, Lys43, Lys123, Lys124, Lys219, Phe166, Asp125, Trp17, Arg192, Leu31, and His23. There are twelve prominent hydrogen bond contacts with four different amino acids: Gln217, Lys124, Lys123, Thr168, Gly171, Glu20, Glu144, Leu31, Gln160, Arg192, Val212, and Lys43. The (MDGP, **1**) compounds (**5–7**) have a high electron density due to the additional benzene ring in the molecule, resulting in the highest binding scores. These data reveal that adding hetero groups such as –Cl, –Br, and –C(CH_3_)_3_ induced binding affinity fluctuations while adding an aromatic ring molecule and a –OH group boosted binding affinity. The docked pose demonstrated that drug molecules bind in the microbial macromolecular structure’s active region.

**Figure 11:**
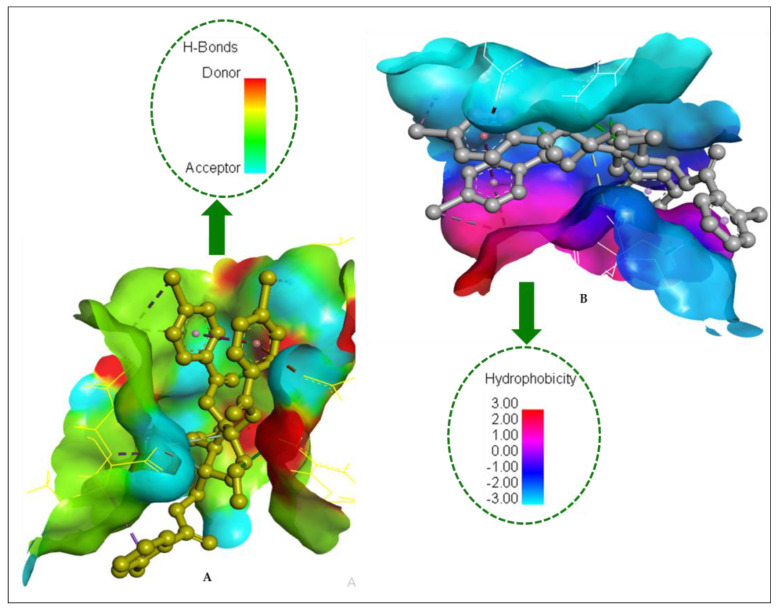
(**A**) Hydrogen bond surface of dengue virus−1 NS2B/NS3 protease compound **5**. (**B**) Hydrophobic bond surface of dengue virus−1 NS2B/NS3 protease with compound **5**.

Along with Phe166, all compounds had the highest π–π interactions with Trp17, Lys43, and Lys124, indicating strong binding with the active site. Some studies imply that Phe166 is the main component of PPS and PPT that makes small molecules accessible at the active site. Due to hydrogen bonding, some compounds (**5–7**) have higher binding energies and modes [58]. The modifications of the -OH group in MDGP, **1** strengthened the π–π interactions with the amino acid chain at the binding site while their polarity improvement caused hydrogen bond interactions. The maximum numbers of H-bonds were observed by compounds **5** and **6** with Glu20, Glu144, Gln160, and Arg192 residues. The H-bond and hydrophobic surfaces of compound (**5**) with dengue virus-1 NS2B/NS3 protease are represented in Figure 9. It was observed from the docking study of all the (MDGP, **1**) compounds with both targets that the molecules are generally surrounded by the abovementioned residues, suggesting that molecules may prevent the microbial activities of the target. The hydrogen bond surface and the hydrophobic bond surface of dengue virus-1 NS2B/NS3 protease with compound **5** are presented in Figure 11.

### 2.15. Molecular-Dynamics (MD) Simulations

**Figure 12:**
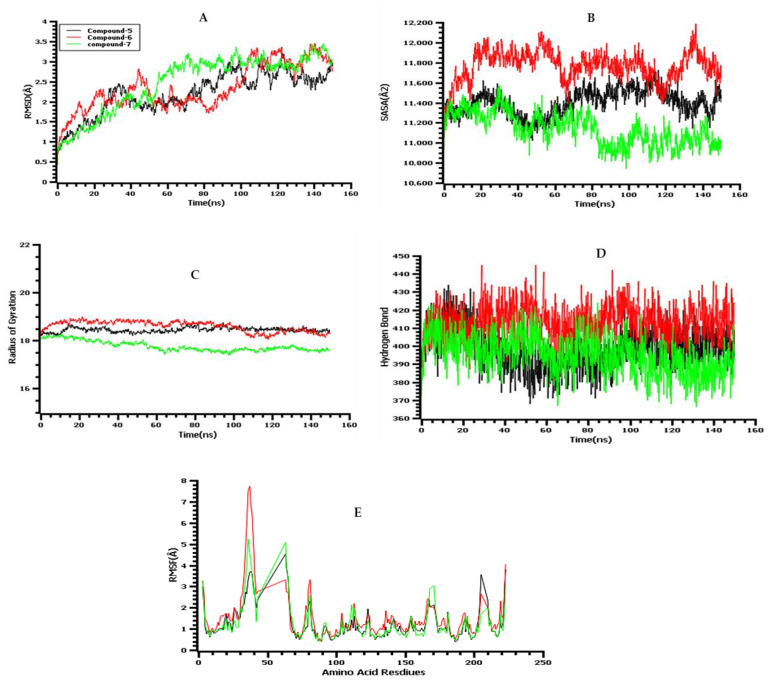
The molecular-dynamics simulation trajectories from 150 ns simulation time, here (**A**) root mean square deviation of the C-alpha atoms; (**B**) solvent accessible surface area of the docked complex; (**C**) radius of the gyration of the complexes; (**D**) hydrogen bond of the complexes and (**E**) root mean square fluctuation.

A molecular-dynamics simulation examined the docked complex’s binding stability. To determine the docked complex binding rigidity, root mean square deviations from the C-alpha atoms of simulated complexes were studied. Figure 12A indicates that compounds **5**, **6**, and **7** had an initial upward trend, which indicates initial flexibility. All three complexes reached the stable state and did not fluctuate after 60 ns until the rest of the simulation periods, which indicated the overall stability of the complexes. The solvent-accessible surface area (SASA) of the simulated complexes was also analyzed. It is known that a higher SASA value defines higher flexibility and a lower SASA value indicates the truncated nature of the complexes. Figure 12B indicates that compound **7** possesses a reduced surface area after 60 ns upon ligand binding. The other two complexes exhibit similar binding patterns after 60 ns to the rest of the simulation time. The radius of gyration (Rg) profile of the simulated complexes defines the labile nature of the complexes, where a higher Rg value is related to a more mobile nature, whereas a lower Rg value is related to the stable conformations of the complexes. Figure 12C indicates that the three complexes have stable and steady trends in Rg and do not overfluctuate [59]. The hydrogen bond of the simulated complexes defines the stability of the drug–protein complexes, whereas all three complexes showed a steady hydrogen bond trend in the simulations (Figure 12D). The root mean square fluctuations (RMSF) of the complexes determine amino acid residue flexibility. Figure 12E shows that the complexes are stable because their maximal residues have lower RMSF.

### 2.16. ADMET Profile and Drug Likeness

ADMET computations compared the absorption, metabolism, and toxicity of all MDGP, **1** molecule. Table 11 shows that all drugs had excellent absorption. If log Kp exceeds −2.5 cm/h, a molecule scarcely penetrates the skin. Skin permeability (Kp) of (MDGP, **1**) compounds ranges from −2.032 to −2.811 cm/h (<−2.5) from Table 11. Thus, all analogs penetrated the skin well. The pkCSM predicts log Papp values > 0.90 cm/s for high Caco-2 permeability. As shown in Table 11, the (MDGP, 1) compounds have poor Caco-2 permeability (log Papp). Table 11 shows that (MDGP, **1**) molecules are soluble [60].

As per the findings of Pires et al. [61], VDss is categorized as low if it falls below 0.71 L/kg (log VDss < −0.15) and high if it exceeds 2.81 L/kg (log VDss > 0.45). The results revealed that the (MDGP, **1**) compounds have VDss values ranging from −0.473 to 0.223, except for two compounds (**6** and **7**), which showed a VDss value < −0.15. Central nervous system medications require blood–brain partitioning and brain distribution. LogBB < −1 molecules are brain poor. From Table 12, MDGP analog logPS (central nervous system (CNS) permeability) ranges from −3.344 to −3.036, which is less than −3. Thus, compounds (**2**–**7**) cannot enter the CNS. From Table 12, (MDGP, **1**) substances had log(CLtot) values from 0.057 to 1.874 mL/min/kg. These values predict compound excretion.

In addition, Table S3 shows that all the analogs do not affect or inhibit all the enzymes, except CYP3A4 for compounds **3**, **6,** and **7**. Therefore, it may be predicted that the other analogs may be metabolized by the P450 enzyme. Table S4 shows that MDGP compounds are fatal only at very large doses due to their high LD50 values (2.04 to 2.30).

### 2.17. Calculation of QSAR and pIC_50_

QSAR and pIC50 were calculated using MLR (multiple linear regression) equations [62]. Our research shows that the total QSAR and pIC50 inquiry value meets all standards and different substances have different values. The QSAR and pIC50 ranged from 3.91 (compound **4**) to 6.25 (compound **6**) (Table 13). The approximate pIC50 value suggests that these newly identified compounds may be physiologically effective against gram-positive and gram-negative bacteria and pathogenic fungi.

## 3. Materials and Methods

### 3.1. Reagents and Instrumentation

Unless otherwise stated, Aldrich reagents were used as supplied. Uncorrected electrothermal melting points were measured in England. A Buchi rotary evaporator (W. Germany) with a bath temperature below 40 °C evaporated under decreased pressure. A Bruker spectrospin spectrometer (Germany) at the BCSIR Laboratories in Dhaka acquired 400 MHz and 100 MHz ^1^H NMR and ^13^C NMR spectra for solutions in deuteriochloroform (CDCl_3_) unless specified (internal Me4Si). Thin layer chromatography (t.l.c) on Kieselgel GF254-detected spots by spraying the plates with 1% H_2_SO_4_ and heating at 150–200 °C until coloration occurred. Column chromatography used silica gel G_60_.

### 3.2. Synthesis of (MDGP, 1) Analogs

A solution of methyl *β***-**d-galactopyranoside (**1**) (100 mg, 0.515 mmol) in dry dimethylformamide (DMF) (3 mL)/TEA (0.15 mL) was cooled to −5 °C and treated with 1.1 molar equivalents of 2-bromobenzoyl chloride (121.8 mg) with continuous stirring by maintaining 0 °C for 6–7 h. Stirring was continued overnight at room temperature. The reaction mixture was continuously stirred at the same temperature for 6 h. The progress of the reaction was monitored by TLC (CH_3_OH-CHCl_3_, 1:6). After stirring at room temperature overnight, the solvent was removed to give a semisolid mass, which was then applied to column chromatography. Initial elution with n-C_6_H_14_ removed the contaminated compounds, and further elution with CH_3_OH-CHCl_3_ (1:6) furnished the title compound, 2-bromobenzoyl derivative (**2**) (180 mg), as a crystalline solid.

Methyl 6-*O*-(2-bromobenzoyl)-*β*-d-galactopyranoside (**2**): Color white crysttaline solid; Yield 92%; m.p. 101–102 °C; (*R_f_* = 0.52); FTIR: 1724 (C=O), 3404~3507 cm^−1^ (br) (-OH); ^1^H NMR (400 MHz, CDCl_3_): δ_H_ 7.81 (1H, d, *J* = 7.6 Hz, Ar-H), 7.63 (1H, d, *J* = 7.4 Hz, Ar-H), 7.31 (2H, m, Ar-H), 5.10 (1H, d, *J* = 8.1 Hz, H-1), 4.52 (1H, dd, *J* = 11.0 and 6.4 Hz, H-6a), 4.50 (1H, dd, *J* = 11.0 and 6.6 Hz, H-6b), 4.19 (1H, d, *J* = 3.6 Hz, H-4), 4.00 (1H, dd, *J* = 3.1 and 10.2 Hz, H-3), 3.87 (1H, dd, *J* = 8.1 and 10.3 Hz, H-2), 3.76 (1H, m, H-5), 3.16 (3H, s, 1-OC*H*_3_); ^13^C NMR (100 MHz, CDCl_3_): δ_C_ 179.0 (2-Br.C_6_H_4_*C*O-), 136.3, 132.4, 130.9, 129.9, 126.5, 125.5 (2-Br. *C*_6_H_4_CO-), 104.1 (C-1), 77.2 (C-2), 77.0 (C-4), 75.2 (C-3), 69.1 (C-5), 63.0 (C-6), 57.0 (1-O*C*H_3_); LC–MS [M + 1]^+^ 378.14; Calcd. For C_14_H_17_O_7_Br: C, 44.55%, H, 4.54%; Found: C, 44.56%, H, 4.56%.

### 3.3. General Procedure for the Synthesis of (2-Bromobenzoyl) Analogs ***3***–***7***

Compound **2** (111.3 mg, 0.30 mmoL) in dry DMF (3 mL) and TEA (0.15 mL) was stirred and cooled to 0 °C. It was then mixed with lauroyl chloride (0.33 mL, 5.0 molar eq.) and stirred for 6 h. Traditional work-up, as described earlier for compound **2**, followed by chromatographic purification (CH_3_OH-CHCl_3_, 1:6, as an eluent), gave lauroate **3** (247.21 mg).

Methyl 6-*O*-(2-bromobenzoyl)-2,3,4-tri-*O*-lauroyl-*β*-d-galactopyranoside (**3**): Color light white; Yield 90%; m.p. 107–108 °C; (*R_f_* = 0.51); FTIR: ν_max_ 1715 (C=O) cm^−1^. ^1^H NMR (400 MHz, CDCl_3_): δ_H_ 7.82 (1H, d, *J* = 7.6 Hz, Ar-H), 7.65 (1H, d, *J* = 7.4 Hz, Ar-H), 7.35 (2H, m, Ar-H), 5.01 (1H, d, *J* = 3.5 Hz, H-1), 4.96 (1H, m, H-2), 4.91 (1H, t, *J* = 9.6 Hz, H-3), 4.51 (1H, t, *J* = 9.4 Hz, H-4), 4.31 (1H, dd, *J* = 2.2 and 12.2 Hz, H-6b), 4.0 (1H, dd, *J* = 4.7 and 10.1 Hz, H-6a), 3.84 (1H, m, H-5), 3.19 (3H, s, 1-OC*H*_3_), 2.36 (6H, m, 3 × CH_3_(CH_2_)_9_C*H*_2_CO-), 1.65 (6H, m, 3 × CH_3_(CH_2_)_8_C*H*_2_CH_2_CO-), 1.27 (48H, m, 3 × CH_3_(C*H*_2_)_8_CH_2_CH_2_CO-), 0.89 (9H, m, 3 × C*H*_3_(CH_2_)_10_CO-); ^13^C NMR (100 MHz, CDCl_3_): δ_C_ 179.0 (2-Br.C_6_H_4_*C*O-), 172.5, 172.4, 172.3 {3 × CH_3_(CH_2_)_10_*C*O*-*}, 135.5, 132.4, 130.9, 129.9, 126.5, 125.5 (2-Br.*C*_6_H_4_CO-), 104.1 (C-1), 77.2 (C-2), 77.1 (C-4), 75.2 (C-3), 69.4 (C-5), 63.0 (C-6), 57.4 (1-O*C*H_3_), 34.3, 34.1 (×2), 31.9 (×3), 29.5 (×3), 29.4, 29.3 (×2), 29.2 (×3), 29.1, 25.0 (×2), 24.3, 22.6 (×3), 22.6, 22.5 (×3), 21.7, 21.6, 20.0 (×2) {3 × CH_3_(*C*H_2_)_10_CO-}, 14.1, 14.0, 13.9 {3 × *C*H_3_(CH_2_)_10_CO-}; LC–MS [M + 1]^+^ 924.93; Calcd. For C_50_H_83_O_10_Br: C, 64.94%, H, 9.06%; Found: C, 64.93%, H, 9.08%.

A similar reaction and purification procedure was applied to prepare compound **4** (myristoyl derivative, 339.4 mg), compound **5** (3-chlorobenzoyl derivative, 161.3 mg), compound **6** (4-chlorobenzoyl derivative, 190. 0 mg), and compound **7** (4-*t*-butylbenzoyl derivative, 167.5 mg).

Methyl 6-*O*-(2-bromobenzoyl)-2,3,4-tri-*O*-myristoyl-*β*-d-galactopyranoside (**4**): Color light white crystalline solid; Yield 70%, m.p. 154–155 °C; (*R_f_* = 0.50); FTIR: ν_max_ 1700 cm^−1^ (C=O); ^1^H NMR (400 MHz, CDCl_3_): δ_H_ 7.81 (1H, d, *J* = 7.6 Hz, Ar-H), 7.63 (1H, d, *J* = 7.4 Hz, Ar-H), 7.31 (2H, m, Ar-H), 5.18 (1H, d, *J* = 3.4 Hz, H-1), 5.08 (1H, dd, *J* = 3.5 and 10.1 Hz, H-2), 4.93 (1H, m, H-3), 4. 88 (1H, t, *J* = 9.7 Hz, H-4), 4.11 (1H, dd, *J* = 4.6 and 11.4 Hz, H-6a), 4.0 (1H, m, H-6b), 3.95 (1H, m, H-5), 3.27 (3H, s, 1-OC*H*_3_), 2.24 {6H, m, 3 × CH_3_(CH_2_)_11_C*H*_2_CO-}, 1.23 {66H, m, 3 × CH_3_(C*H*_2_)_11_CH_2_CO-}, 0.88 {9H, m, 3 × C*H*_3_(CH_2_)_12_CO-}; ^13^C NMR (100 MHz, CDCl_3_): δ_C_ 179.0 (2-Br.C_6_H_4_*C*O-), 172.5, 172.4, 172.4 {3 × CH_3_(CH_2_)_12_*C*O-}, 136.3, 132.4, 130.9, 129.9, 126.5, 125.5 (2-Br.*C*_6_H_4_CO-), 104.1 (C-1), 77.2 (C-2), 77.1 (C-4), 75.0 (C-3), 69.3 (C-5), 63.1 (C-6), 57.0 (1-O*C*H_3_), 34.3, 34.3, 34.1 (×2), 31.8, 31.9 (×2), 29.5 (×2), 29.4, 29.3, 29.3 (×2), 29.2 (×3), 29.1, 25.0 (×2), 24.9, 24.9, 22.6 (×3), 22.6, 22.6 (×3), 22.6 (×3), 21.7, 21.6, 20.0 (×2), 20.0 {3 × CH_3_(*C*H_2_)_12_CO-}, 14.1, 14.0, 13.9 {3 × *C*H_3_(CH_2_)_12_CO-}; LC–MS [M + 1]^+^ 1009.08; Calcd. For C_56_H_95_O_10_Br: C, 66.66%, H, 9.50%; Found: C, 66.67%, H, 9.52%.

Methyl 6-*O*-(2-bromobenzoyl)-2,3,4-tri-*O*-(3-chlorobenzoyl)-*β*-d-galactopyranoside (**5**): Color white needles; Yield 77%; m.p. 136–137 °C; (*R_f_* = 0.51); FTIR: ν_max_ 1707 (-CO) cm^−1^. ^1^H NMR (400 MHz, CDCl_3_): δ_H_ 8.01 (3H, m, Ar-H), 7.85 (1H, d, *J* = 7.5 Hz, Ar-H), 7.82 (3H, m, Ar-H), 7.60 (1H, d, *J* = 7.2 Hz, Ar-H), 7.47 (3H, m, Ar-H), 7.37 (2H, m, Ar-H), 7.34 (3H, m, Ar -H), 5.32 (1H, d, *J* = 8.0 Hz, H-1), 5.21 (1H, dd, *J* = 8.0 and 10.2 Hz, H-2), 5.21 (1H, dd, *J* = 3.1 and 10.3 Hz, H-3), 4.72 (1H, d, *J* = 3.7 Hz, H-4), 4.51 (1H, dd, *J* = 11.1 and 6.6 Hz, H-6a), 4.22 (1H, dd, *J* = 11.2 and 6.8 Hz, H-6b), 4.03 (1H, m, H-5), 3.28 (3H, s, 1-OC*H*_3_); ^13^C NMR (100 MHz, CDCl_3_): δ_C_ 179.1 (2-Br.C_6_H_4_*C*O-), 167.3, 165.2, 164.2 (3 × 3-Cl.C_6_H_4_.*C*O-), 136.3, 132.3, 130.3, 129.6, 126.5, 125.6 (2-Br.*C*_6_H_4_CO-), 131.9 (×3), 131.5 (×2), 131.1 (×3), 129.4 (×4), 129.0 (×4), 128.8 (×2) (3 × 3-Cl.*C*_6_H_4_.CO-), 104.3 (C-1), 77.1 (C-2), 77.1 (C-4), 75.2 (C-3), 69.1 (C-5), 63.3 (C-6), 57.1 (1-O*C*H_3_); LC–MS [M + 1]^+^ 793.65; Calcd. For C_35_H_26_O_10_Br.Cl: C, 52.99%, H, 3.31%; Found: C, 52.98%, H, 3.32%.

Methyl 6-*O*-(2-bromobenzoyl)-2,3,4-tri-*O*-(4-chlorobenzoyl)-*β*-d-galactopyranoside (**6**): Color light white; Yield 77%, m.p. 184–185 °C; (*R_f_* = 0.52); FTIR: ν_max_ 1711 (-CO) cm^−1^. ^1^H NMR (400 MHz, CDCl_3_): δ_H_ 8.01 (6H, m, Ar-H), 7.81 (6H, m, Ar-H), 7.72 (1H, d, *J* = 7.6 Hz, Ar-H), 7.43 (1H, d, *J* = 7.4 Hz, Ar-H), 7.30 (2H, m, Ar-H), 5.79 (1H, d, *J* = 3.6 Hz, H-1), 5.53 (1H, dd, *J* = 3.6 and 10.0 Hz, H-2), 5.0 (1H, m, H-3), 4.76 (1H, t, *J* = 9.6 Hz, H-4), 4.22 (1H, m, H-6a), 4.0 (1H, t, *J* = 10.2 Hz, H-6b), 3.55 (1H, m, H-5), 3.18 (3H, s, 1-OC*H*_3_); ^13^C NMR (100 MHz, CDCl_3_): δ_C_ 178.7 (2-Br.C_6_H_4_*C*O-), 167.2, 166.7, 164.2 (3 × 3-Cl.C_6_H_4_.*C*O-), 136.2, 132.3, 130.4, 129.5, 126.3, 125.6 (2-Br.*C*_6_H_4_CO-), 132.3 (×3), 131.4 (×2), 131.3 (×3), 129.3 (×4), 129.1 (×4), 128.2 (×2) (3 × 3-Cl.*C*_6_H_4_.CO-), 104.1 (C-1), 77.3 (C-2), 77.3 (C-4), 75.1 (C-3), 69.4 (C-5), 63.2 (C-6), 57.1 (1-O*C*H_3_); LC–MS [M + 1]^+^ 793.65; Calcd. For C_35_H_26_O_10_Br.Cl: C, 52.99%, H, 3.31%; Found: C, 52.98%, H, 3.33%.

Methyl 6-*O*-(2-bromobenzoyl)-2,3,4-tri-*O*-(4-t-butylbenzoyl)-*β*-d-galactopyranoside (**7**): Color white; Yield 53%. m.p. 122–123 °C; (*R_f_* = 0.53); FTIR: ν_max_ 1718 (-CO) cm^−1^. ^1^H NMR (400 MHz, CDCl_3_): δ_H_ 8.04 (6H, m, 3 × Ar-H), 7.79 (1H, d, *J* = 7.4 Hz, Ar-H), 7.62 (1H, d, *J* = 7.4 Hz, Ar-H), 7.59 (6H, m, 3 × Ar-H), 7.31 (2H, m, Ar-H), 5.20 (1H, d, *J* = 8.2 Hz, H-1), 5.71 (1H, dd, *J* = 8.1 and 10.5 Hz, H-2), 5.68 (1H, dd, *J* = 3.1 and 10.6 Hz, H-3), 5.12 (1H, d, *J* = 3.5 Hz, H-4), 5.0 (1H, dd, *J* = 11.1 and 6.2 Hz, H-6a), 4.81 (1H, dd, *J* = 11.0 and 6.3 Hz, H-6b), 4.08 (1H, m, H-5), 3.28 (3H, s, 1-OC*H*_3_), 1.25, 123, 122{27H, 3 × s, 3×(C*H*_3_) _3_C-}; ^13^C NMR (100 MHz, CDCl_3_): δ_C_ 179.0 (2-Br.C_6_H_4_*C*O-), 174.4, 174.2, 174.1 {3×(CH_3_)_3_CC_6_H_4_CO-}, 135.8, 132.5, 130.4, 129.5, 126.5, 125.3 (2-Br.*C*_6_H_4_CO-), 132.4 (×3), 132.4 (×2), 132.4, 130.9 (×3), 129.9 (×3), 126.5 (×3), 125.5 (×3) {3×(CH_3_)_3_CC_6_H_4_CO-}, 104.3 (C-1), 77.3 (C-2), 77.1 (C-4), 75.3 (C-3), 69.3 (C-5), 63.2 (C-6), 57.1 (1-O*C*H_3_), 35.6, 35.5, 35.4 {(×3)(CH_3_)_3_CC_6_H_4_CO-}, 13.6 (×3), 13.6 (×3), 13.4 (×3) {(×3)(CH_3_)_3_CC_6_H_4_CO-}; LC–MS [M+1]^+^ 858.66; Calcd. For C_47_H_53_O_10_Br: C, 65.76%, H, 6.23%; Found: C, 65.77%, H, 6.22%.

### 3.4. Microorganisms

Five human pathogenic bacteria and two plant pathogenic fungi were used in the current study. The details about these strains are shown in Table S5. All microorganisms were obtained from the Department of Microbiology, Faculty of Biological Science, University of Chittagong, Bangladesh. The isolates were kept frozen at a temperature of −20 °C until they were needed. When the bacterial strains were needed, they were grown on Mueller–Hinton agar medium for 18 h at a temperature between 30–37 °C. Similarly, the fungal strains were grown on Sabouraud dextrose agar medium for a period of 5 to 7 days at 25 °C [63].

### 3.5. Antibacterial Activity

The methodology employed to determine the antibacterial properties of the synthesized compounds (each compound dissolved in dimethyl sulfoxide 7% DMSO) involved the use of the disc diffusion approach. This was done following a previously described method with slight modifications [64]. First, an inoculum was obtained from a bacterial colony and diluted to a concentration of 0.5 McFarland standard in sterile saltwater containing 0.9% NaCl. The suspension was then transferred to a sterile tube and allowed to settle for 5 min, after which the top homogeneous solution was transferred to another sterile tube. The concentration of the solution was adjusted to a 0.5 McFarland turbidity standard to obtain an inoculum of approximately 10^6^ CFU/mL.

### 3.6. Determination of MIC and MBC

To determine the minimum inhibitory concentration (MIC) of the synthesized compounds against the tested bacterial strains [63,64], a dilution method was employed. The objective was to establish the lowest concentration of the compound at which approximately 90% of bacterial growth was inhibited. To this end, a bacterial suspension of 300 μL at a concentration of 0.5 McFarland standard was seeded into individual tubes of 9 mL nutrient broth, with each tube receiving 1 mL of different concentrations of the synthesized compound at twofold serial dilutions and 7% DMSO, which served as the negative control. The tubes were then kept at 37 °C for a whole night. The MIC and MBC values were determined by using the naked eye to compare the turbidity of the tube to that of a reference medium with a 0.5 McFarland value.

### 3.7. Antifungal Activity

The food poisoning method [65] with potato dextrose agar (PDA) as the culture medium was used to test the antifungal action. Twenty milliliters of 45 °C sterilized, melted potato dextrose agar medium (PDA) was placed into sterile 70-mm glass Petri plates. The “poisoned food” method [64] was used to determine how the synthesized MDGP (**1**) derivatives affected the growth of the fungal mycelium.

### 3.8. Cytotoxic Activity Evaluation

The brine shrimp lethality assay (BSLA) was utilized to evaluate the toxicity of the MDGP analogs [66]. Each vial contained 5 mL of NaCl solution and 20 μL of MDGP analogs dissolved in DMSO. Vials A, B, C, and D had volumes of 4, 8, 16, and 32 μL, respectively. Each vial was inoculated with 10 brine shrimp nauplii at three different concentrations. A control test was conducted using ten nauplii in 5 mL of saltwater. The vials were incubated at ambient temperature for 24–48 h. Following incubation, the vials were examined under magnification and enumerated to ascertain the number of viable specimens. Concentrations exhibit a mean nauplii mortality rate. There were no fatalities in the control group.

### 3.9. Structure–Activity Relationship (SAR)

A structure–activity relationship (SAR) analysis was performed to identify the active portion of the synthesized molecule. This popular technology is often used in drug design according to Hunt [67] and Kim’s [68] membrane permeation concept.

### 3.10. PASS Prediction and Bioactivity

The online pass website (http://www.pharmaexpert.ru/passonline/) (accessed on 23 March 2023), which is the most accurate site for predicting the bioactivity of synthesized MDGP, **1** compound, was used to obtain the pass prediction data (Pa > Pi value) [69]. PASS outcomes are revealed by Pa (probability for active molecule) and Pi (probability for inactive molecule). With potentialities, the Pa and Pi scores vary in the range of 0.00 to 1.00 and, usually, Pa + Pi ≠ 1, as these potentialities are predicted freely. Biological actions with Pa > Pi are only thought of as probable for a selected drug molecule. In the present study, the Molinspiration online server (https://www.molinspiration.com/cgi-bin/properties) (accessed on 25 March 2023) was utilized to analyze the drug-like properties of lead compounds. The Molinspiration cheminformatics engine allows for the fast prediction of biological activity and virtual screening of large collections of molecules and the selection of molecules with the highest probability to show biological activity. Then, the compound structures were drawn and changed into their smile forms using the SwissADME free online weblink (http://www.swissadme.ch) (accessed on 26 March 2023) to find the antimicrobial spectrum using the PASS tool. In particular, the Pa > Pi value was examined for its antiviral, antifungal, and antibacterial effects.

### 3.11. Geometry DFT Optimization

In computer-aided drug design, quantum mechanical methods are often used to predict thermal, molecular orbital, and molecular electrostatic potential qualities. Using the Gaussian 09 tool [70], all structures’ geometries were improved and changed further. Density functional theory (DFT) with Becke’s three-parameter hybrid model (B) [71] and Lee, Yang, and Parr’s (LYP) correlation functional [72]. The first optimization of all chemicals was performed in the gas phase. For each of the MDGP analogs, the HOMO-LUMO energy gap, hardness (η), and softness (S) were calculated from the energies of the frontier HOMO and LUMO as reported, considering Parr and Pearson’s interpretation of DFT and Koopman’s theorem [73] on the correlation of chemical potential (µ), electronegativity (χ), and electrophilicity (ω) with HOMO and LUMO energy (ε). The following equations were used to calculate global chemical reactivity by analyzing molecular orbital features [74].

Gap(∆ε)=εLUMO−εHOMO

$$\eta =\frac{\left[\varepsilon LUMO-\varepsilon HOMO\right]}{2}$$

$$S=\frac{1}{\eta }$$

$$µ=\frac{\left[\varepsilon LUMO+\varepsilon HOMO\right]}{2}$$

$$\chi =-\frac{\left[\varepsilon LUMO+\varepsilon HOMO\right]}{2}$$

$$\omega =\frac{\mu 2}{2\eta }$$

### 3.12. Protein Selection and Molecular Docking

The structure of the dengue virus 1 NS2B/NS3 protease (PDB ID: 3L6P) was found in the protein databank library [75]. Using PyMol (version 1.3) software packages [76], all heteroatoms and water molecules were removed. Using Swiss-PdbViewer (version 4.1.0), the protein’s energy was minimized. Then, molecular docking modeling [77] was performed using the PyRx application (version 0.8), imagining the target protein as a macromolecule and the MDGP-1 compounds as ligands. The protein and ligands were input by converting the pdb format to pdbqt, and the AutoDock Tools of the MGL software package were used to perform this job. In AutoDockVina, the size of the grid box was maintained at (47.7033, 64.6084, and 51.6510 Å) along the X, Y, and Z axes and was centered using the following dimension, −2.021 × 2.164 × 6.319 and grid spacing 0.061 × 0.061 was used to cover the active site along with the essential residues within the binding pocket. After docking, the structures of both the macromolecule and ligand were saved in pdbqt format and Accelrys Discovery Studio (version 4.1) was employed to explore the results of docking and to predict the nonbonding interactions among the (MDGP, **1**) analogs and amino acid chains of the receptor protein [78,79]. Using a Lig plot and a Ramachandran plot (Figure S6) to check the validity of the target receptor, more than 90% of the residues were in the allowed area.

### 3.13. Molecular-Dynamics Simulation

The molecular-dynamics simulation study was performed with the help of the AMBER14 force field [80] and the YASARA dynamics [81] software package. The steepest gradient algorithms and the simulated annealing method (5000 cycles) were used to obtain starting energy levels as low as possible [82]. The simulations were run with a time step of 2.0 fs [83]. The simulation paths were saved every 100 ps and the end run was performed for 150 ns. Root mean square deviations (RMSD), root mean square fluctuations (RMSF), radius of gyrations (Rg), solvent accessible surface area (SASA), and hydrogen bonds were all calculated using the simulation paths [84,85].

### 3.14. Pharmacokinetic and Drug-Likeness Prediction

ADMET properties are one of the most significant aspects of drug molecules and are described as pharmacokinetic properties. For this reason, the best-identified analogs were evaluated using pkCSM [61] for their in silico pharmacokinetic parameters, including intestinal absorption, blood–brain barrier, metabolism, clearance, and toxicity.

## 4. Conclusions

In this research, a series of (MDGP, **1**) compounds were synthesized and analyzed for their in vitro antimicrobial, cytotoxicity, and in silico properties. The insertion of various aliphatic and aromatic groups into the (MDGP, **1**) structures can significantly improve their biological activity. The synthesized compounds were more effective against gram-positive bacteria than gram-negative bacteria. The examined compounds also showed potential efficacy against all fungal strains. In particular, the study showed that benzoyl compounds (**5–7**) had better pharmacokinetic and biological profiles and could be more effective against bacteria and fungi. Molecular docking was used to explain these findings because it showed that MDGP compounds have promising antimicrobial effects. With the dengue virus-1 NS2B/NS3 protease, MDGP compounds **2**–**7** had positive interactions and binding energies when they were bound to it. The ability of the compounds (**5–7**) to fight against the target was very effective in silico studies. The molecular electrostatic potential study showed where the ligand had the most negative and positive surface areas. This helped predict where the best places for hydrogen bonding sites would be. This finding was strongly supported by MD simulations at 150 ns, which showed that the docked complex was stable in its binding in the trajectory analysis. Additionally, most of the designed molecules had better kinetic factors and still followed all of the rules for drugs and therapeutics.

## Figures and Tables

**Table 1:** Structures of synthesized (MDGP, **1**) analogs **2**–**7**.

Entry	Chemical Structure	Mol. Formula
**2**	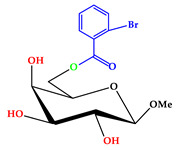	C_14_H_17_O_7_Br
**3**	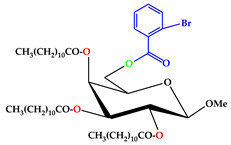	C_50_H_83_O_10_Br
**4**	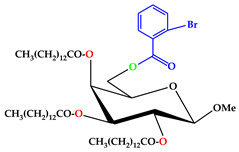	C_56_H_95_O_10_Br
**5**	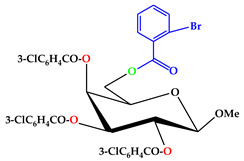	C_35_H_26_O_10_Br.Cl
**6**	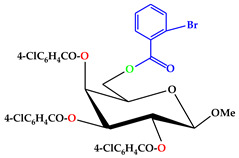	C_35_H_26_O_10_Br.Cl
**7**	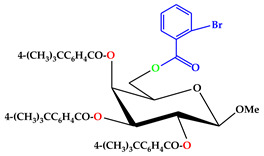	C_47_H_53_O_10_Br

**Table 2:** ^1^H NMR and ^13^C NMR assignments were obtained from HSQC and HMBC experiments.

Position	δ_H_ (ppm) (*J* Hz)	(HSQC) δ_C_ (ppm)	HMBC
Ar-H	7.81 (d, *J* = 7.6)	136.3	H : Ar
Ar-H	7.63 (d, *J* = 7.4)	132.4	H : Ar
H-1	5.10 (d, *J* = 8.1)	104.1	H : 2, OCH_3_
H-6a	4.52 (dd, *J* = 11.0 and 6.4)	63.1	H : 5, CO
H-6b	4.50 (dd, *J* = 11.0 and 6.6)	63.0	H : 5, CO
H-4	4.19 (d, *J* = 3.6)	77.0	H : 3, 5
H-3	4.00 (dd, *J* = 3.1 and 10.2)	75.2	H : 2, 4
H-2	3.87 (dd, *J* = 8.1 and 10.3)	77.2	H : 1, 3
H-5	3.76 (m)	69.1	H : 4, 6a, 6b
1-O*CH_3_*	3.16 (s)	57.0	H : 1
2-Br.C_6_H_4_CO-		179.0	H : 6a, 6b

**Table 3:** FTIR, LC–MS, and physicochemical properties of the tested (MDGP, **1**) compounds (**2**–**7**).

Entry	Solvent and *R_f_*	FTIR (KBr, ν_max_) cm^−1^	LC–MS [M + 1]^+^	mp. (°C)	Yield (%)	Found (Calculated)	
						%C	%H
**2**	CH_3_OH-CHCl_3_ (1:6) (*R_f_* = 0.51)	1724 (C=O), 3404~3507 cm^−1^ (br) (-OH)	378.14	101–102	92	44.56 (44.55)	4.56 (4.54)
**3**	CH_3_OH-CHCl_3_ (1:7) (*R_f_* = 0.52)	1715 (C=O)	924.93	107–108	90	64.93 (64.94)	9.08 (9.06)
**4**	CH_3_OH-CHCl_3_ (1:6) (*R_f_* = 0.53)	1700 (C=O)	1009.08	154–155	70	66.67 (66.66)	9.52 (9.50)
**5**	CH_3_OH-CHCl_3_ (1:5) (*R_f_* = 0.51)	1707 (-CO)	793.65	136–137	77	52.98 (52.99)	3.32 (3.31)
**6**	CH_3_OH-CHCl_3_ (1:5) (*R_f_* = 0.54)	1711 (-CO)	793.65	184–185	77	52.98 (52.99)	3.33 (3.31)
**7**	CH_3_OH-CHCl_3_ (1:6) (*R_f_* = 0.50)	1718 (-CO)	858.66	122–123	53	65.77 (65.76)	6.22 (6.23)

**Table 4:** Antibacterial susceptibility by the tested analogs.

	Diameter of Inhibition Zones (In mm)				
Entry	*B. subtilis* (G + ve) (ATCC 6633)	*S. aureus* (G + ve) (BTCC 19)	*E. coli* (G − ve) (ATCC 8739)	*S. typhi* (G − ve) (AE 14612)	*P. aeruginosa* (G − ve) (ATCC 9027)
**1**	NI	NI	NI	NI	NI
**2**	NI	10.50 ± 0.2	NI	11.25 ± 0.2	* 15.00 ± 0.1
**3**	* 13.00 ± 0.1	* 15.50 ± 0.3	11.00 ± 0.2	* 12.25 ± 0.1	11.25 ± 0.3
**4**	NI	NI	NI	NI	NI
**5**	NI	NI	NI	NI	* 18.25 ± 0.3
**6**	* 12.75 ± 0.1	11.50 ± 0.3	11.25 ± 0.2	* 13.00 ± 0.3	11.50 ± 0.1
**7**	7.75 ± 0.1	NI	8.50 ± 0.1	9.00 ± 0.3	7.50 ± 0.3
Azithromycin	** 18.25 ± 0.2	** 17.50 ± 0.2	** 17.25 ± 0.2	** 18.0 ± 0.1	** 18.5 ± 0.3

All experimental triplicate values are shown. Asterisks (*) and double asterisks (**) indicate significant inhibition (*p* < 0.05). NI = No inhibition.

**Table 5:** Antifungal potentiality of the synthesized analogs in (%) of inhibition.

Entry	% Inhibition of Fungal Mycelial Growth in mm	
	*Aspergillus niger* (ATCC 16404)	*Aspergillus flavus* (ATCC 204304)
**1**	NI	NI
**2**	* 72.88 ± 1.0	* 85.66 ± 0.9
**3**	* 64.83 ± 1.1	* 84.02 ± 1.3
**4**	* 71.19 ± 1.0	NI
**5**	48.73 ± 1.1	* 81.97 ± 1.0
**6**	* 78.81 ± 1.0	* 81.97 ± 1.2
**7**	46.61 ± 0.7	NI
Nystatin	** 66.7 ± 1.1	** 65.2 ± 1.2

* Significant inhibition; ** Reference antibiotic, NI: no inhibition.

**Table 6:** Prediction of antimicrobial activity of the (MDGP, **1**) compounds.

Diameter of Inhibition Zone In mm						
Entry	Antiviral		Antibacterial		Antifungal	
	Pa	Pi	Pa	Pi	Pa	Pi
**1**	0.511	0.021	0.414	0.036	0.374	0.091
**2**	0.607	0.011	0.502	0.024	0.440	0.032
**3**	0.411	0.107	0.487	0.022	0.473	0.024
**4**	0.393	0.131	0.487	0.022	0.473	0.024
**5**	0.571	0.045	0.533	0.024	0.504	0.048
**6**	0.554	0.129	0.539	0.062	0.567	0.048
**7**	0.548	0.066	0.551	0.039	0.536	0.21

**Table 7:** Determination of the drug-likeness score of (MDGP, **1**) compounds through the molinspiration cheminformatics online server.

Entry	GPCR Ligand	Ion Channel Modulator	Kinase Inhibitor	Nuclear Receptor Ligand	Protease Inhibitor	Enzyme Inhibitor
**1**	−0.13	−0.24	−0.17	−1.19	−0.71	0.88
**2**	0.04	0.23	0.09	0.47	0.21	0.55
**3**	0.19	−0.46	−0.23	−0.58	−0.23	0.11
**4**	−0.21	−0.91	−0.49	−0.77	−0.19	−0.06
**5**	−0.05	−1.09	0.63	−1.11	0.27	0.24
**6**	0.27	0.42	0.14	0.51	0.27	0.24
**7**	0.36	0.40	−0.11	0.34	0.26	0.23

**Table 8:** Energy (eV) of HOMO, LUMO, Gap (∆), hardness (*η*), softness (*S*), chemical potential (*µ*), and electronegativity (*χ)* and electrophilicity (*ω*) of (MDGP, **1**) compounds.

Entry	HOMO	LUMO	Gap (∆_Ԑ_)	*η*	*S*	*µ*	*χ*	*ω*
**1**	−6.021	−0.391	5.630	2.815	0.355	3.206	−3.206	2.317
**2**	−6.100	−0.863	5.237	2.618	0.381	3.481	−3.481	2.314
**3**	−6.233	−0.925	5.308	2.654	0.376	3.579	−3.579	2.413
**4**	−6.589	−0.897	5.692	2.846	0.351	3.743	−3.743	2.461
**5**	−5.148	−0.203	4.945	2.472	0.404	2.675	−2.675	1.447
**6**	−6.236	−0.758	5.478	2.739	0.365	3.497	−3.497	2.232
**7**	−5.850	−0.726	5.124	2.562	0.390	3.288	−3.288	2.019

**Table 9:** Binding energy of the (MDGP, **1**) compounds against dengue virus-1 NS2B/NS3 protease (3L6P).

Entry	3L6P (kcal/mol)
**1**	−5.5
**2**	−6.6
**3**	−6.1
**4**	−5.1
**5**	−8.1
**6**	−8.0
**7**	−8.3

**Table 10:** Nonbonding interaction data of (MDGP, **1**) compounds against dengue virus-1 NS2B/NS3 protease (3L6P).

Entry	Bond Category	Residues in Contact	Interaction Type	Distance (Å)
**1**	H	Gly201	CH	2.4942
	H	Phe180	CH	2.4973
	H	Gly183	CH	2.0541
	H	Thr184	CH	2.5129
	H	Tyr211	CH	1.9715
	H	Lys181	C	3.6387
**2**	H	Gln217	CH	2.0536
	H	Gln217	CH	2.1626
	H	Gln217	CH	2.3156
	H	Lys124	C	3.5204
	H	Thr168	C	3.5964
	Hydrophobic	Ile215	PA	5.2676
**3**	H	Lys123	CH	2.1638
	H	Gly171	C	3.4559
	Hydrophobic	Val204	A	5.0692
	Hydrophobic	Ala141	A	3.8337
	Hydrophobic	Lys123	PA	4.0691
	Hydrophobic	Phe166	PA	4.5004
**4**	H	Gly171	C	3.73352
	Hydrophobic	Lys219	A	3.7839
	Hydrophobic	Val173	A	5.4094
	Hydrophobic	Lys124	A	4.8909
**5**	H	Glu20	CH	2.6152
	H	Leu31	CH	2.1156
	H	Gln160	CH	2.0824
	H	Arg192	CH	1.9956
	H	Arg192	CH	2.5099
	H	Arg192	CH	2.0967
	Electrostatic	Glu19	PAn	4.1395
	Hydrophobic	Trp17	PPT	5.2745
	Hydrophobic	Arg192	A	3.9082
	Hydrophobic	Leu31	A	4.4797
	Hydrophobic	Arg192	PA	4.0916
	Hydrophobic	Leu31	PA	4.5535
**6**	H	Glu144	CH	2.1561
	H	Gln160	CH	2.9897
	H	Gln160	CH	2.9917
	H	Arg192	CH	2.7495
	H	Lys43	C	3.7800
	H	Lys43	C	3.6630
	Electrostatic	Glu19	PAn	3.6453
	Hydrophobic	Leu31	PS	3.4595
	Hydrophobic	Lys43	PS	3.7162
	Hydrophobic	Leu31	A	4.2217
	Hydrophobic	Ile30	A	4.7386
	Hydrophobic	His23	PA	4.2129
	Hydrophobic	Ile30	PA	3.8409
**7**	H	Val212	C	3.2112
	Electrostatic	Asp125	PAn	4.3311
	Hydrophobic	Val173	PS	3.9352
	Hydrophobic	Val204	PS	3.8277
	Hydrophobic	Phe166	PPS	4.8680
	Hydrophobic	Ala214	A	4.3635
	Hydrophobic	Val173	A	5.0337
	Hydrophobic	Val204	A	5.0532
	Hydrophobic	Phe166	PA	5.0407
	Hydrophobic	Lys124	PA	4.7050
	Hydrophobic	Ala214	PA	5.1764
	Hydrophobic	Lys123	PA	5.3628

CH = Conventional Hydrogen Bond; C = Carbon Hydrogen Bond; A = Alkyl; PA = Pi-Alkyl; PS = Pi-sigma; PAn = Pi-anion; PPS = Pi-Pi stacked; PPT = Pi-Pi T-shaped.

**Table 11:** Prediction of in silico absorption of (MDGP, **1**) compounds.

Entry	Water Solubility (log mol/L)	Caco-2 Permeability	Intestinal Absorption	Skin Permeability
**1**	−3.01	−0.658	59.069	−3.118
**2**	−4.450	0.360	77.541	−2.032
**3**	−4.759	0.407	81.201	−2.237
**4**	−5.151	0.593	86.001	−2.370
**5**	−5.257	0.421	91.379	−2.561
**6**	−5.369	0.664	93.907	−2.791
**7**	−5.857	0.487	96.325	−2.811

**Table 12:** Prediction in silico of distribution and execution of (MDGP, **1**) compounds.

Entry	Distribution			Execration	
	Vdss	BBB Permeability	CNS Permeability	Total Clearance	Renal OCT2 Substrate
**1**	−0.204	−0.877	−4.087	0.321	No
**2**	−0.315	−1.114	−3.324	1.874	No
**3**	−0.473	−1.137	−3.344	1.850	No
**4**	0.223	−1.265	−3.231	1.379	No
**5**	−0.417	−1.301	−3.122	1.198	No
**6**	−0.079	−1.210	−3.036	0.057	No
**7**	−0.055	−1.354	−3.047	0.089	No

**Table 13:** Data of QSAR.

Entry	Chiv5	(bcutm1)	(MRVSA9)	(MRVSA6)	(PEOEVSA5)	GATSv4	PIC50
**1**	0.494	2.343	0.000	0.00	0.00	0.92	4.78
**2**	1.112	2.884	7.299	0.00	58.270	0.99	4.25
**3**	1.710	4.917	15.109	73.32	68.807	1.07	4.24
**4**	2.873	4.816	28.237	81.41	77.896	1.15	3.91
**5**	4.027	3.201	35.342	93.71	80.100	1.21	5.66
**6**	5.630	4.663	55.317	98.22	107.636	1.23	6.25
**7**	6.449	4.371	39.441	107.11	144.675	1.37	6.07

## Data Availability

Data are available in the article and the Supplementary Materials.

## Supplementary Materials

The following supporting information can be downloaded at: https://www.mdpi.com/article/10.3390/ph16070998/s1, Figure S1: Spectra; Figure S2–S5: Antimicrobial; Figure S6: Alignment and Ramachandran plot of dengue virus 1 NS2B/NS3. Table S1: MIC and MBC values; Tables S2–S4: In silico; Table S5: Name of the pathogenic microorganisms.
