# Supplementary material for: Galactoside-Based Molecule Enhanced Antimicrobial Activity through Acyl Moiety Incorporation: Synthesis and In Silico Exploration for Therapeutic Target
Source: Pharmaceuticals (Basel). 2023 Jul 13;16(7):998. doi: 10.3390/ph16070998 (PMC10385442; doi:10.3390/ph16070998)
Supplement: Supplementary file 1 [file pharmaceuticals-16-00998-s001.zip › pharmaceuticals-2444689-supplementary.pdf]

pharmaceuticals

MDPI

Supplementary Material

Galactoside-Based Molecule Enhanced Antimicrobial Activity through Acyl Moiety Incorporation: Synthesis and *In Silico* Exploration for Therapeutic Target

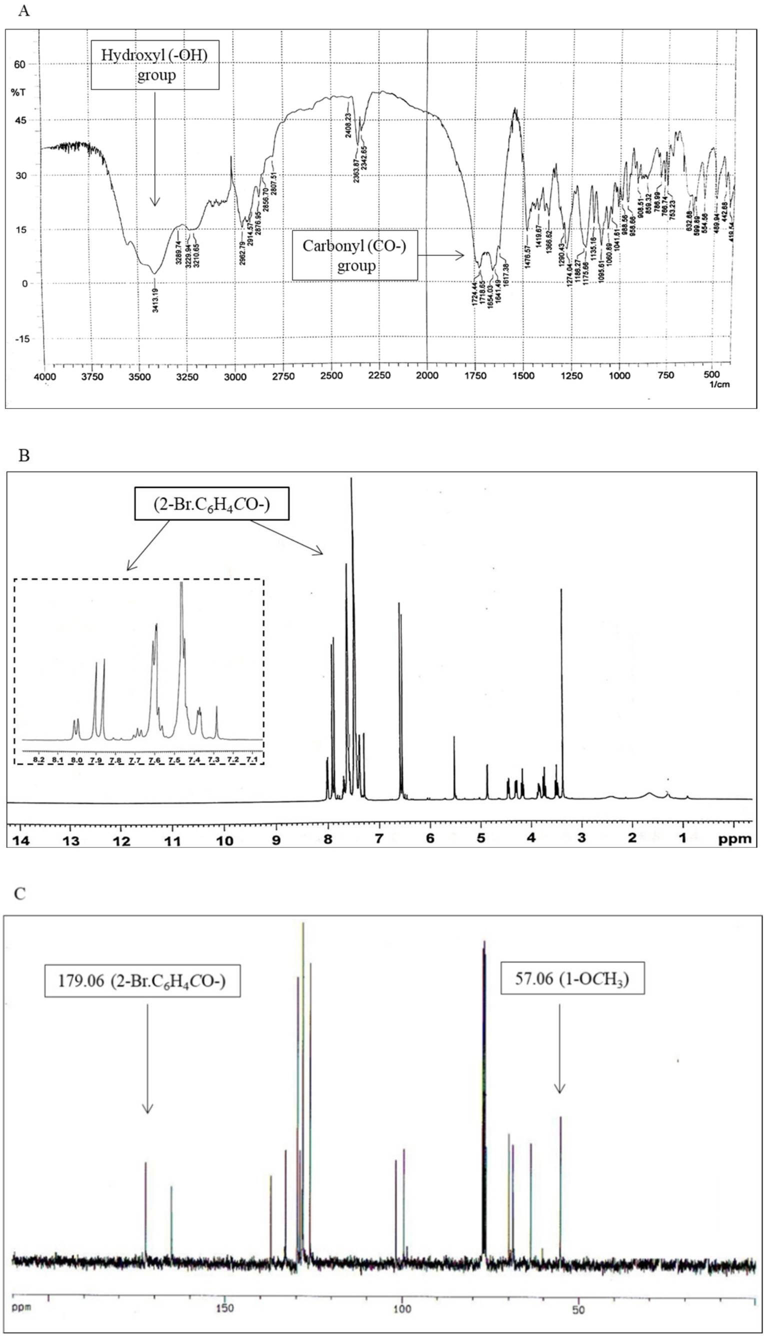

Figure S1. (A) FTIR, (B) ^{1}H NMR and (B) ^{13}C NMR spectra of the methyl 6-*O*-(2-bromobenzoyl)-*β*-D-galactopyranoside (**2**).

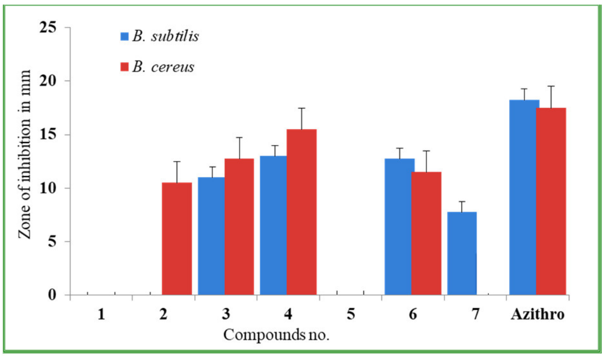

Figure S2. Zone of inhibition observed against gram-positive bacteria by compounds **2-7**.

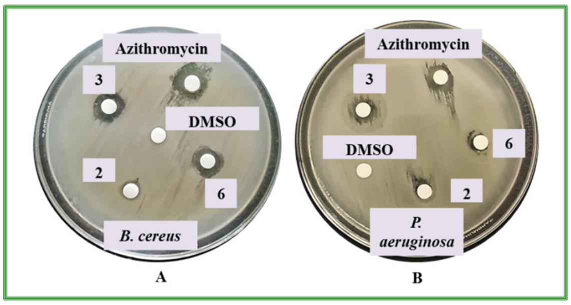

Figure S3. Experimental dishes of the synthesized test compounds **2, 3** and **6** against (A); *B.cereus* and (B); *P. aerruginosa*, Here DMSO = Negative control and Azithromycin = Positive control.

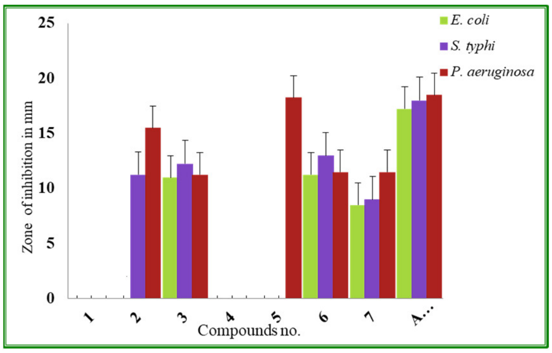

Figure S4. Zone of inhibition observed against gram-negative bacteria by compounds **2-7.**

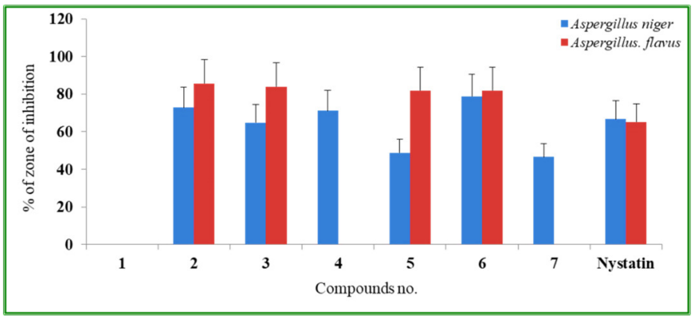

Figure S5. Antifungal activities of the synthesized compounds **2-7.**

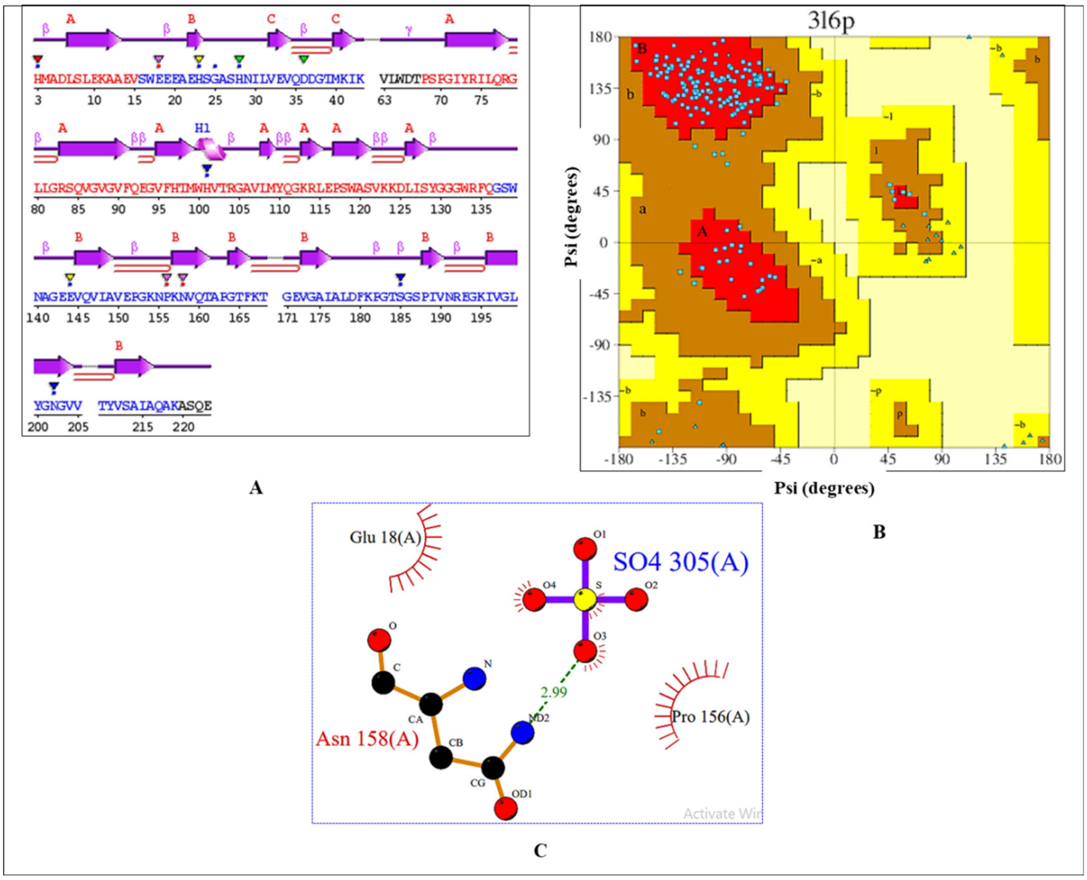

Figure S6. (A): Multiple sequence alignment of closest homologs of dengue virus 1 NS2B/NS3 protease (pdb: 3L6P); (B): Ligplot and (C): Ramachandran plot of dengue virus 1 NS2B/NS3 protease (pdb: 3L6P.

Table S1. The MIC and MBC values in mg/mL of compounds **2** and **3** against tested organisms.

**Name of bacteria**	**MIC (mg/mL)**		**MBC (mg/mL)**	
	**Compound 3**	**Compound 6**	**Compound 3**	**Compound 6**
B. subtilis (ATCC 6633)	8.00	0.50	16.00	16.00
B. cereus (BTCC 19)	8.00	2.00	8.00	8.00
E. coli (ATCC 8739)	2.00	1.00	16.00	8.00
S.typh (AE 14612)*i*	0.125	2.00	8.00	8.00
P. aeruginosa (ATCC 9027)	0.25	8.00	16.00	16.00

Table S2. Molecular formula, molecular weight, electronic energy (*E*), enthalpy (*H*), Gibb's free energy (*G*) in Hartree and dipole moment (*p*, Debye) of (MDGP, **1**) compounds.

**Entry**	**MF**	**MW**	***E***	***H***	***G***	***p***
**1**	$C_7H_{14}O_6$	194.18	-722.2093	-722.2084	-722.2608	4.771
**2**	$C_{14}H_{17}O_7Br$	377.14	-3625.844	-3625.843	-3625.919	5.425
**3**	$C_{50}H_{83}O_{10}Br$	923.93	-5253.279	-5253.278	-5253.479	5.162
**4**	$C_{56}H_{95}O_{10}Br$	1008.8	-5862.524	-5862.523	-5862.671	5.023
**5**	$C_{35}H_{26}O_{10}BrCl_3$	792.65	-6025.296	-6025.295	-6025.426	2.980
**6**	$C_{35}H_{26}O_{10}BrCl_3$	792.65	-6025.289	-6025.288	-6025.419	8.200
**7**	$C_{47}H_{53}O_{10}Br$	857.66	-5121.303	-5121.302	-5121.462	3.463

Table S3. Prediction of *in silico* of metabolism of (MDGP, **1**) compounds.

Entry	Cyp1A2	Cyp2C19	Cyp2D6	Cyp3A4
**1**	No	No	No	No
**2**	No	No	No	No
**3**	No	No	No	Yes
**4**	No	No	No	No
**5**	No	No	No	No
**6**	No	No	No	Yes
**7**	No	No	No	Yes

Table S4. Prediction *in silico* of the toxicity of (MDGP, **1**) compounds.

Entry	Ames toxicity	T. Pyriformis Toxicity	Herg1 inhibition	LD50	Skin sensitisation
**1**	No	0.184	No	2.533	No
**2**	No	0.365	No	2.046	No
**3**	No	0.278	No	2.185	No
**4**	No	0. 278	No	2.185	No
**5**	No	0.158	No	2.308	No
**6**	No	0.107	No	2.291	No
**7**	No	0.126	No	2.317	No

Table S5. Name of the pathogenic microorganisms.

Types of organisms	Strain	Reference
Gram-positive bacteria	*Bacillus subtilis*	ATCC 6633
	Bacillus cereus	BTCC 19
Gram-negative bacteria	*Escherichia coli*	ATCC 8739
	Salmonella typhi	AE 14612
	Pseudomonas aeruginosa	ATCC 9027
Name of the fungi	*Aspergillus niger*	ATCC 16404
	Aspergillus flavus	ATCC 204304
